# Sodium-Dependent Multivitamin Transporter Facilitates Brain and Neuronal Uptake of a Creatine Analogue

**DOI:** 10.1021/acschemneuro.6c00484

**Published:** 2026-09-17

**Authors:** Adéla Králová, Ville Kuorikoski, Landry Anamea, Janne Tampio, Arun Kumar Tonduru, Hengjing Cui, Henri Leinonen, Jaana Rysä, Antti Poso, Tetsuya Terasaki, Aaro Jalkanen, Thales Kronenberger, Kristiina M. Huttunen

**Affiliations:** † School of Pharmacy, Faculty of Health Sciences, 4344 University of Eastern Finland, Kuopio 70211, Finland; ‡ Department of Pharmacology, Faculty of Medicine, 205537 University of Helsinki, Helsinki 00014, Finland; § Interfaculty Institute of Microbiology and Infection Medicine (IMIT), University of Tübingen, and German Center for Infection Research (DZIF), Partner Site Tübingen, Elfriede-Aulhorn-Str. 6, Tübingen 72076, Germany

**Keywords:** sodium-dependent multivitamin transporter (SMVT), creatine, prodrug, transporter-mediated drug delivery, creatine deficiency syndrome, brain delivery

## Abstract

Creatine transporter 1 (CRT1, SLC6A8) is a membrane transporter essential for importing creatine across the blood–brain barrier (BBB) into neurons and glial cells. Loss-of-function mutations in CRT1 result in creatine transporter deficiency syndrome (CTDS), a severe X-linked neurological disorder with no effective treatment. To explore alternative uptake pathways for creatine, a carrier-mediated delivery system utilizing the sodium-dependent multivitamin transporter (SMVT), a potential neuronal target to facilitate creatine brain uptake, was employed. In this study, SMVT was investigated *in silico* and *in vitro* to identify key substrate-binding residues and to functionally characterize the transporter in human glioblastoma (U-87MG) and mouse neuronal cells. Furthermore, four novel SMVT-utilizing creatine derivatives were designed, synthesized, and assessed *in vitro*. The most promising derivative was studied *in vivo* and in a creatine kinase (CK) enzymatic assay and molecular dynamics (MD) simulations to determine its interactions with the ATP/ADP system. The studies confirmed SMVT expression in human glioblastoma cells and mouse neurons, and functional assays demonstrated pH- and concentration-dependent transporter activity in both cell lines. The creatine derivatives were taken up by human glioblastoma cells, and the lead derivative, compound **1** (COMP **1**), demonstrated SMVT-mediated uptake at concentrations below 100 μM and was also taken up by mouse neurons. Finally, COMP **1** accumulated in the mouse brain and showed interactions with the CK system. Collectively, these findings identify SMVT as a potential route for brain uptake of creatine derivatives and support further investigation of SMVT-targeting derivatives in relevant CTDS animal models.

## Graphical abstract

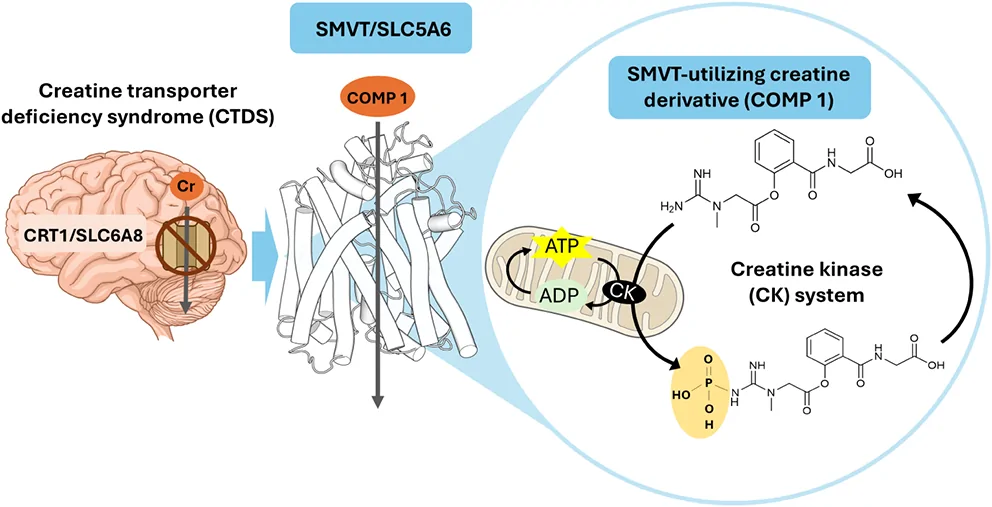

## 1 Introduction

Creatine (N-aminoiminomethyl-*N*-methylglycine) plays a crucial role in intracellular energy metabolism, especially in tissues with high energy demands such as muscle and brain.^1,2^ The system comprising creatine, phosphocreatine (P-creatine), and creatine kinases (CKs) serves as an energy buffer to recycle adenosine triphosphate (ATP), by taking up ATP at its production sites and delivering it to its consumption sites.^3^ Beyond its role in improving muscle function, creatine also exhibits neuroprotective potential through lipid membrane stabilization, antioxidant and antiapoptotic actions, and antiexcitotoxic effects mediated in part by interactions with glutamate and GABA_A_ receptors.^4–6^ The beneficial effects of creatine supplementation have been reported in depression, Huntington’s disease, and traumatic brain injury. Clinical studies have shown the antidepressant potential of creatine, improving symptoms of major depressive disorder (5 g/day, 8-week treatment),^7^ bipolar depression (3–5 g/day, 4-week treatment),^8^ and traumatic brain injury (0.4 g/kg, 6-month treatment).^9,10^ Another clinical study has revealed that, in Huntington’s disease, creatine supplementation (8 g/day, 16-week treatment) suppressed serum levels of 8-hydroxy-2′-deoxyguanosine (8OH2’dG), an indicator of oxidative damage to DNA.^11^

Creatine, supplied from the diet or endogenously synthesized, is transported into the brain by creatine transporter 1 (CRT1, encoded by the *SLC6A8* gene).^12^ CRT1 belongs to the sodium-dependent neurotransmitter family of solute carrier 6 transporters, and in the brain, it is predominantly expressed in neurons, oligodendrocytes, and microcapillary endothelial cells forming the blood–brain barrier (BBB).^13^ Thus, CRT1 is necessary for transporting and regulating the creatine concentration from the systemic circulation across the BBB and further from the brain extracellular space to target sites in brain parenchymal cells.^2^ Mutations in CRT1 cause one of the most common causes of hereditary intellectual disability, creatine transporter deficiency syndrome (CTDS).^14^ CTDS is characterized by brain creatine deficiency and significant developmental delays, including behavioral abnormalities, intellectual disability, and motor dysfunction. While errors in creatine biosynthesis are easily treatable by oral creatine supplementation, an effective CTDS treatment is unavailable because of dysfunctional CRT1 and its inability to transport creatine across the BBB into the brain.^15,16^

In contrast to CRT1, sodium-dependent multivitamin transporter (SMVT, *SLC5A6*) is a broadly substrate-permissive transmembrane protein with strong potential for exploitation in drug delivery.^17^ SMVT is responsible for translocating water-soluble vitamins and essential cofactors such as biotin (vitamin B7 or H), pantothenic acid (vitamin B5), and lipoic acid into the cells.^18^ Importantly, SMVT is abundantly expressed in the human BBB, and its expression in the small intestine supports its use for orally administered prodrugs.^19–21^ Moreover, SMVT has already been utilized to deliver a biotin-conjugated anticancer drug to enhance its cellular permeability and intracellular retention in SMVT-overexpressing cancer cell lines.^18^ Collectively, SMVT represents a potential transporter of interest for central nervous system (CNS)-targeted drug delivery systems, such as prodrugs. By using a prodrug approach, improved delivery and cell selectivity can be achieved, with fewer side effects, as has been demonstrated previously for various other targets.^22–24^

Capitalizing on this concept, we investigated SMVT as a neuronal transporter and evaluated its potential to facilitate creatine delivery across the BBB and into neurons. Four novel SMVT-utilizing creatine derivatives (COMP **1**–**4**) were rationally designed, synthesized, and evaluated (Figure 1). Molecular docking and molecular dynamics (MD) simulations were performed with SMVT to generate potential protein–ligand binding modes to identify lead compounds. The SMVT-utilizing creatine compounds were synthesized, and the *in vitro* accumulation in target cells as well as the *in vivo* pharmacokinetic profile of the most promising compound were evaluated to explore their potential as innovative candidates for neuronal creatine delivery.

**Figure 1:**
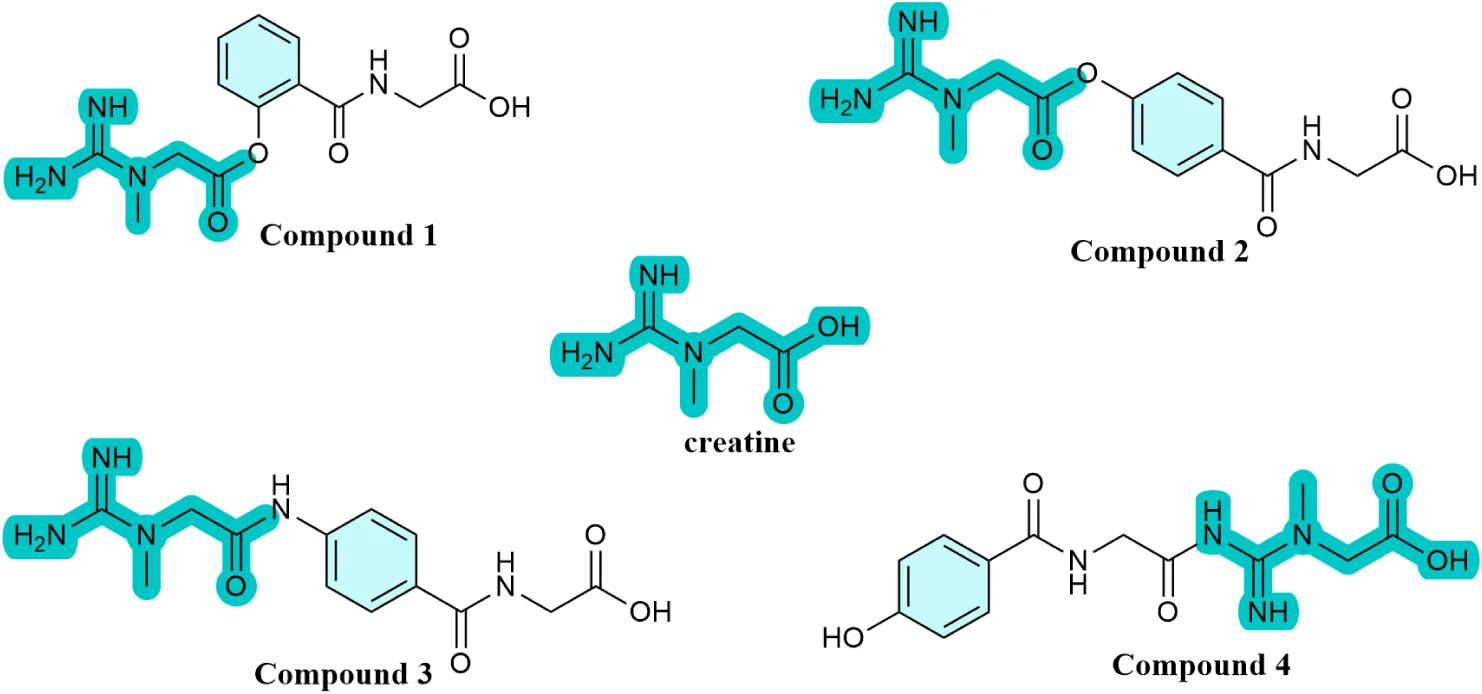
Structures of compounds **1–4** (COMP **1–4**) and their parent drug creatine.

## 2 Results and Discussion

### 2.1 SMVT-Prodrug Simulations Highlight Key Binding-Site Residues

Since no high-resolution structures of human SMVT had been reported when this work was initiated, an AlphaFold protein model was used for computational ligand-binding studies. The model predicts 13 transmembrane helices (TMs) with a large extracellular loop located between TM12 and TM13. SMVT appears to function independently, with the N-terminal tail located extracellularly and the C-terminal tail facing the cytoplasm. The cytoplasmic exit is sealed by TMs 2, 6, 7, and 9, forming a gate homologous to that in SMCT1 (Figure 2A). A buried binding pocket was identified using SiteMap (SiteScore: 1.24, Figure 2B,C), with a volume of 178 Å^3^ and a mixed polar–hydrophobic character. Similar buried binding sites are present in homologous transporters such as sodium-glucose cotransporter 1 (SGLT1) and sodium-coupled monocarboxylate transporter 1 (SMCT1).

**Figure 2:**
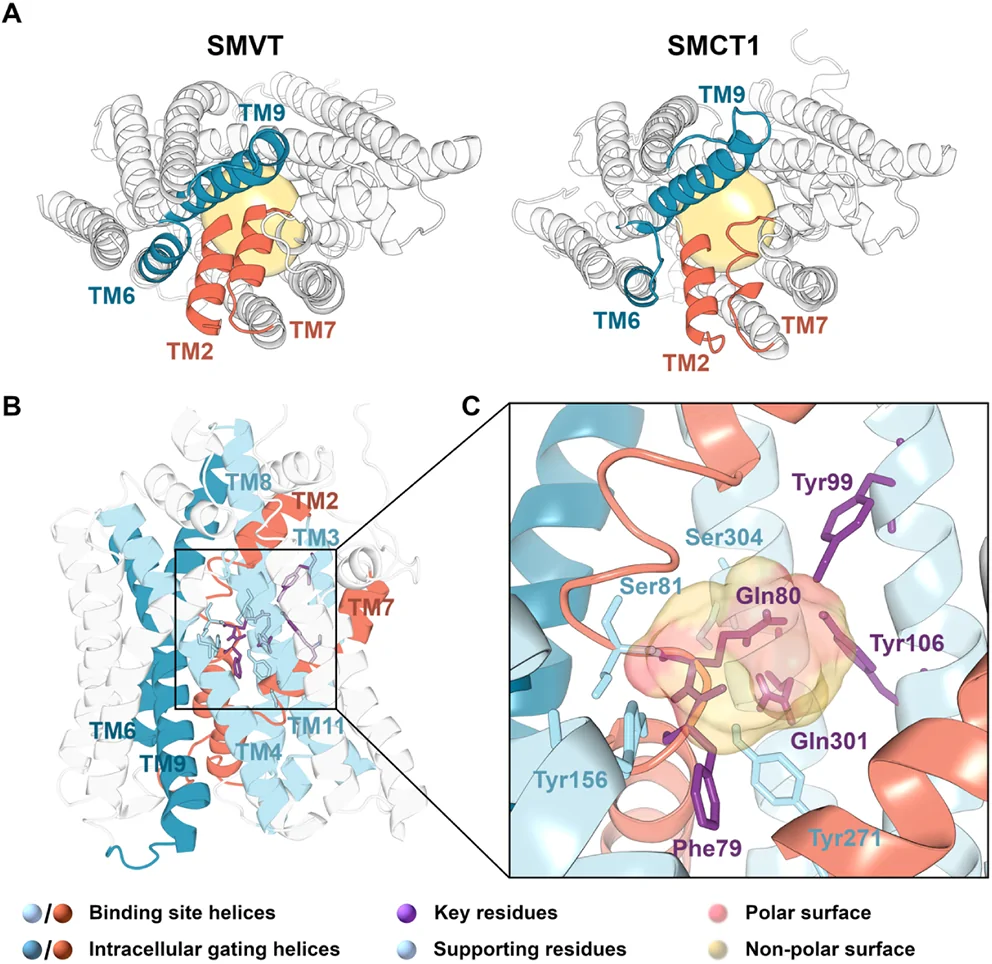
(**A**) Cytosolic view of SMVT compared with its homologue SMCT1. The SMVT structure shows transmembrane helices (TM) 2 and 7 (orange) together with 6 and 9 (blue) forming a gate that occludes the exit pathway from the binding site (yellow). For reference, SMCT1 (PDB: 7SL9) is displayed in an inward-open state with the gate slightly open, using the same color scheme for the corresponding elements. (**B**) Initial SMVT model highlighting the buried pocket residues and the surrounding TMs. Key residues are displayed in purple and supporting residues in light blue. (**C**) Zoomed view of the binding site with the initial volume visualized.

The pocket is located in the internal cavity, composed of TMs 2, 3, 4, 7, 8, and 11. A helical break in TM2 extends into the pocket and positions Phe79 near its center (Figure 2B), a feature also observed in SMCT1 (PDB: 7SL9) and SGLT1 (PDB: 7SL8). This extension of the helical break loop was found to be structurally conserved in SGLT1. However, unlike SMVT’s hydrophobic Phe79, SGLT1 features the polar residue asparagine at the same position. In contrast, SMCT1 also features the hydrophobic residue phenylalanine at this position, like SMVT, but lacks the extended loop structure present in both SMVT and SGLT1. Additionally, flexible docking indicated that the site residues in TM7 and TM11 exhibit the most conformational freedom to accommodate large ligands.

To identify the critical interacting residues contributing to the binding of creatine derivatives, we performed MD simulations. Using a modified MD setup as published before,^25^ preliminary screening was performed, simulating a set of compounds with inhibitory effects that were previously tested on SMVT-mediated biotin uptake.^19^ This led to the selection of biotin, pantothenic acid, lipoic acid, PAH, prostaglandin E_2_, and ketoprofen for further investigation in longer simulations. Across these simulations, a consistent set of interacting residues emerged: Phe79, Gln80, Tyr99, Tyr106, and Gln301, termed key residues. Notably, all these residues populated one side of the pocket in the TMs 2, 3, and 8 (Figure 2B,C). Extending into the pocket, Phe79 (TM2) contributed to binding through stable polar interactions with its backbone and further hydrophobic interactions with its side chain. Residue contributions were quantified as the fraction of simulation time during which the ligand contact was maintained.

### 2.2 Design and Assessment of Creatine Prodrugs

After the different compounds had been studied, *para*-aminohippuric acid (PAH) was identified as a potential promoiety structure for new prodrug designs. It formed stable interactions with all the most frequently interacting residues Supporting Information, Figure S1D) and adapted a binding pose similar to that of the natural substrate, biotin. Additionally, PAH featured a reactive site on the amino group’s nitrogen for conjugating a parent drug to the structure. These features highlighted PAH as a promising carrier for prodrug designs. Building on these features, prodrugs were designed by conjugating creatine with PAH, resulting in four derivatives: COMP **1–4** (Figure 1).

MD trajectories of the natural substrates and COMP **1–4** were clustered based on ligand RMSD, and representative poses from the most populated clusters were analyzed. Comparison with the original docked poses showed that ligand orientations were largely maintained, while surrounding residues adapted through side-chain rearrangements and TM backbone shifts. This highlights that docking provides reasonable initial poses, whereas MD captures the dynamic adaptations underlying binding.

MD simulations of SMVT with COMP **1–4** or its natural substrates corroborated the well-established need for a free carboxylic acid group for SMVT-mediated transport.^26^ The carboxyl group formed frequent polar interactions with several binding site residues, influencing the binding pose heavily (Figure 3A,B). Considering only the carboxyl-specific interactions, substrate binding was mediated primarily by the key residues other than Phe79 (Supporting Information Figures S2, S3).

**Figure 3:**
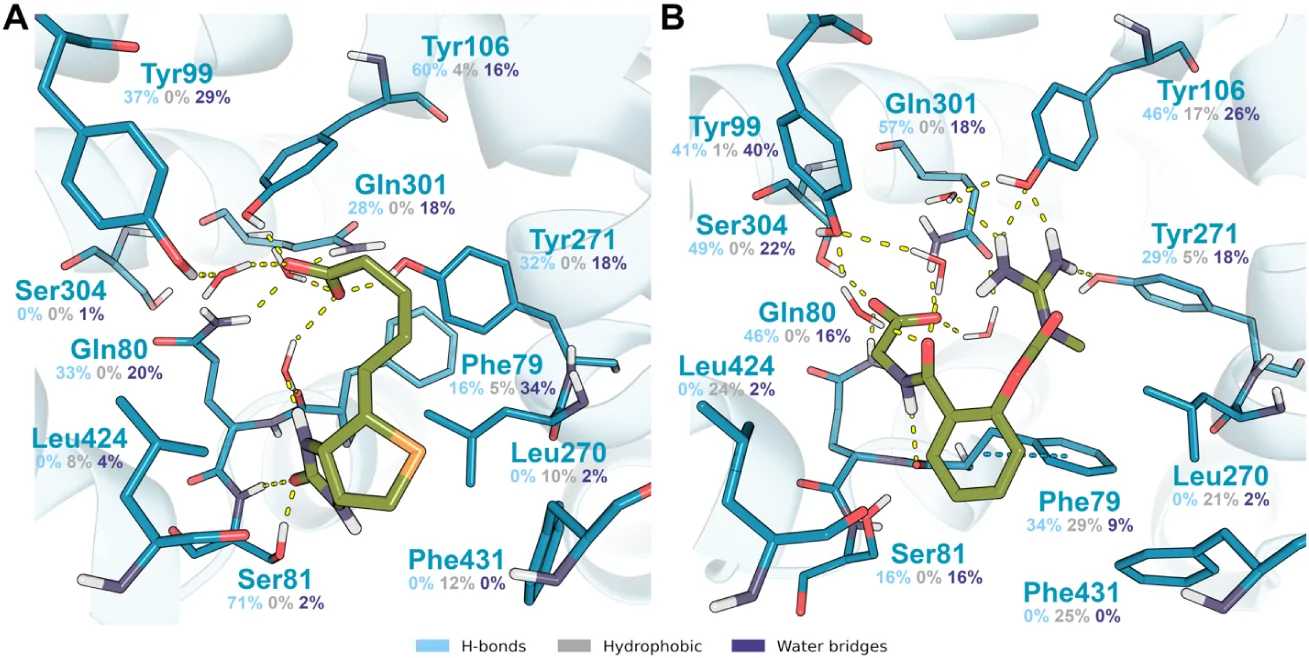
Inset view of the SMVT binding site highlighting interactions with (**A**) biotin and (**B**) COMP **1**, color-coded with residue-specific interaction frequencies detailed by interaction type.

For COMP **1**, binding was dominated by seven residues that contributed for more than 50% of the simulation time: the five key residues and two additional residues, Tyr271 and Ser304 (Figure 3B; Supporting Information Figure S4A). COMP **1** adopted a bent conformation, positioning its aromatic ring near a hydrophobic region formed by Phe79 (TM2), Leu270 (TM7), Leu424 (TM11), and Phe431 (TM11), while its carboxyl and guanidine groups alternately interacted with the persistent residue set (Supporting Information Figure S5).

For COMPs **2** and **3**, the main contributors were Phe79, Gln80, Tyr99, Tyr106, Gln214, and Ser304. COMP **3** adopted a predominantly linear conformation, extending its carboxyl group into a hydrophilic cavity and interacting with Gln80, Gln214, and Ser304 for 88%, 87%, and 73% of the simulation time, respectively (Supporting Information Figure S3), whereas COMP **2** adopted a bent conformation while engaging the same residues for 52%, 50%, and 63%, respectively. Ligand RMSD analysis indicated lower positional fluctuation for COMP **2** than for COMP **3** (Supporting Information Figure S6). COMP **4** relied on a similar residue set, except that its interaction with Tyr99 was replaced by one with Gln301, and it did not adopt a distinct conformation compared with COMP **2** and COMP **3**. Across these compounds, the phenolic hydroxyl of COMP **4** and the noncarboxyl terminals of COMP **2** and COMP **3** did not form stable interactions within the binding site.

These observations highlighted key differences and similarities among COMP **1–4** relative to the natural substrates. The significant contributors to the binding mode, Phe79, Gln80, Tyr106, and Ser304, were conserved across all four compounds, while additional contacts with Tyr99, Gln214, and Gln301 varied in a compound-dependent manner. Notably, Tyr271 was specific to COMP **1** among the tested compounds and was also involved in interactions with the natural substrates.

The observed differences in the adopted conformations were likely due to the specific conjugation types and attachment points of each compound. The planar amide bond in COMP **3** favored a linear conformation, while the flexible ester bond in COMP **2** allowed the molecule to bend. Comparison of the ligand RMSD suggested that COMP **2** adopted a more stable binding pose than COMP **3**, possibly because of this bent conformation. Among all four compounds, COMP **4** was the only compound without an open guanidine moiety, while the ortho substitution in COMP **1** enabled a unique arrangement within the binding site. The two terminals of COMP **1** were oriented toward the same region, which allowed them to interact with the key residues targeted by natural substrates (Figure 3A,B).

In contrast, COMP **2**–**4** interacted with a slightly different set of residues, prominently featuring Gln214, which was not involved in natural substrate binding. Notably, all four compounds differed from the natural substrates by forming interactions with Ser304, a contact unique to these ligands. The phenolic hydroxyl of COMP **4** and the noncarboxyl terminals of COMP **2** and **3** failed to form stable interactions, unlike certain natural substrates, such as biotin with the ketone carbonyl (Figure 3A) or pantothenic acid with the terminal hydroxyl, which consistently engaged the site. COMP **1**, however, could stabilize the pose with its benzene ring. Overall, these results suggest that COMP **1** exhibits the most favorable binding profile, mimicking the key interactions observed with the natural substrates. Nevertheless, all four originally designed compounds were synthesized to enable the experimental validation of these predictions.

### 2.3 Synthesis and Stability of Compounds

The SMVT-targeted compounds (COMP **1–4**) were synthesized by using conventional organic chemistry techniques as previously described.^27^ Creatine was coupled with SMVT utilizing hippuric acids to afford the following creatine derivatives (Scheme 1) by gradually optimizing step-by-step the reaction conditions on a microwave synthesizer. The coupling reaction was achieved with the aid of 1-ethyl-3-(3-(dimethylamino)­propyl)­carbodiimide hydrochloride (EDC·HCl) and N,N-diisopropylethylamine (DIPEA) in the *N*,*N*-dimethylformamide (DMF) solution. The final compounds were chromatographically purified and characterized by NMR, MS, and HPLC.

**Scheme 1:**
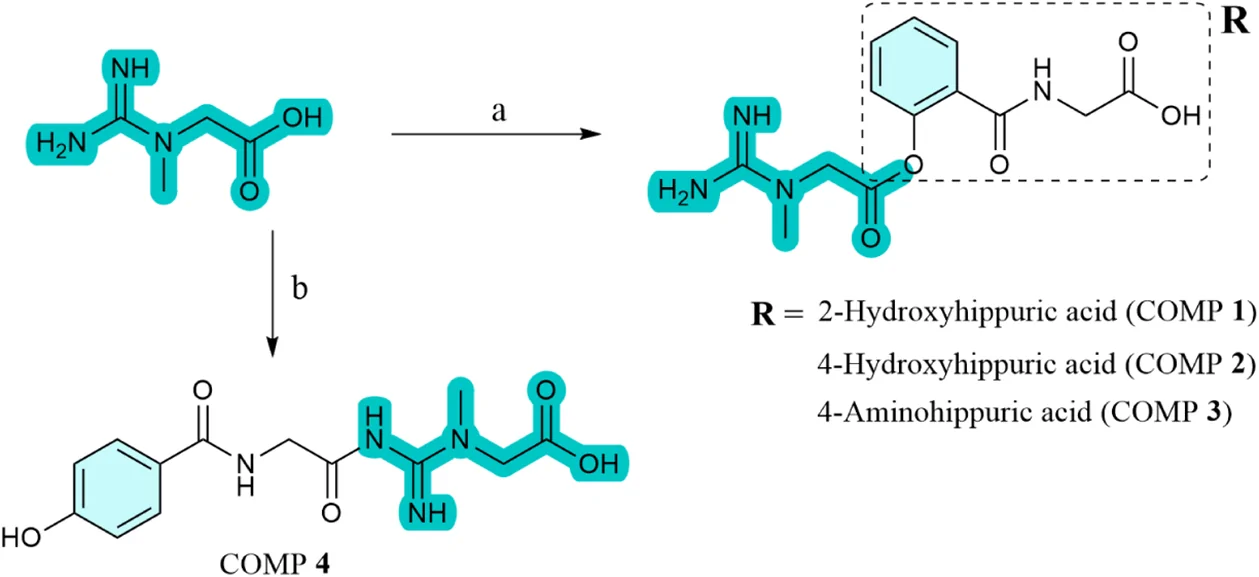
Synthetic Reaction for Creatine-Coupled Derivatives^a^

a Reagents and conditions: (a) creatine, EDCI•HCl, DIPEA, DMF, hippuric acids, microwave, 100 °C, 2 h, 28–33%; (b) 4-hydroxyhippuric acid, EDCI·HCl, DIPEA, DMF, creatine, microwave, 100 °C, 30 min and 2 h, 16%.

The chemical and enzymatic stability of COMP **1–4** (100 μM) were investigated in 0.1 M NaOH, 50 mM Tris-HCl, pH 7.4, human plasma, mouse serum, mouse liver S9 fraction, and mouse brain cell homogenates (astrocytes and microglial cells) at 37 °C for selected time points. All compounds studied showed good chemical stability, with >64% of compounds remaining after 1 h and 24 h in NaOH and Tris-HCl, respectively. Compounds **2–4** also exhibited high stability in all studied biological media (>62%, Table 1). Lower stability was observed with ester-bonded COMP **1** in human plasma, mouse serum, and mouse brain cell homogenates. Nonetheless, its enzymatic metabolism was slow, with half-lives exceeding 2 h in all matrices, and the greatest stability was observed in human plasma (Table 2). Notably, the released creatine or creatinine was not detected in any matrices.

**Table 1:** Chemical and Enzymatic Stability of Compounds **1–4** in 0.1 M NaOH, 50 mM Tris-HCl, pH 7.4, Human Plasma, Mouse Serum, Mouse Liver S9 Fraction, as well as in Mouse Brain Homogenate (Presented as Remaining Percentages, %; Mean ± SD, n = 3)

compound	0.1 M NaOH	50 mM Tris-HCl pH 7.4	human plasma	mouse serum	mouse liver S9	mouse brain cell homogenate
**1**	77.36 ± 3.52	98.74 ± 3.92	40.49 ± 5.15	27.08 ± 3.68	55.01 ± 10.01	28.37 ± 5.36
**2**	77.36 ± 23.05	70.74 ± 9.63	63.90 ± 7.08	81.32 ± 1.91	93.90 ± 3.40	95.82 ± 7.92
**3**	95.14 ± 6.26	87.70 ± 24.86	98.34 ± 6.12	79.18 ± 25.95	99.60 ± 3.67	62.14 ± 10.80
**4**	64.57 ± 6.15	64.54 ± 15.83	92.71 ± 6.13	80.07 ± 5.94	99.50 ± 8.39	97.73 ± 19.77

**Table 2:** Enzymatic Half-Lives of Compound **1** in Human Plasma, Mouse Serum, and Mouse Brain Cell Homogenate, Presented as Half-Lives, t_1/2_ (Min; Mean ± SD, n = 3)

compound	matrix	t1/2 (min)
**1**	Human plasma	334.42 ± 0
**1**	Mouse serum	156.31 ± 12.08
**1**	Mouse brain cell homogenate	226.93 ± 20.67

### 2.4 SMVT Is Functionally Expressed in Human Glioblastoma Cells and Mouse Neurons

To limit the number of mouse pups required for primary neuronal isolation, a suitable immortalized screening cell line was used for early-stage characterization of SMVT, system validation, and compound screening. Based on our results, the human glioblastoma cell line U-87MG exhibited relatively high SMVT expression. Expression levels were compared with the plasma membrane housekeeping protein, sodium–potassium adenosine triphosphatase (Na^+^/K^+^ ATPase), and the l-type amino acid transporter 1 (LAT1) dimer, consisting of light (LAT1) and heavy (4F2hc) subunits, a well-established transporter target in CNS drug delivery.^24,28^ As seen in Figure 4A and Table 3, SMVT expression in U-87MG cells was almost 3-fold higher (0.73 ± 0.31 fmol/μg of protein) than the expression of LAT1 transporter (0.26 ± 0.10 fmol/μg of protein), and 42-fold smaller than Na^+^/K^+^ ATPase. Since the expression of SMVT was proved, U-87MG cells were used to evaluate SMVT functionality using a specific SMVT-utilizing substrate (biotin) under various conditions—different pH and concentration, and in the presence/absence of SMVT competitive substrates/inhibitors (Figure 4B–D). Results revealed that biotin uptake gradually diminishes with increasing pH (from 0.020 ± 0.006 nmol/min/mg protein at pH 4.5 to 0.006 ± 0.001 nmol/min/mg protein at pH 8.5), indicating that the process is pH-dependent. This pH dependence of SMVT was in accordance with previous results proven in different cell types: human corneal epithelial (HCEC), retinal pigment epithelial (RPE, D407), and retinoblastoma (Y-79) cells.^29,30^ The concentration-dependent cellular uptake of biotin (5–200 μM) further demonstrated efficient biotin transport into U-87MG cells. The maximum transport capacity (*V*_max_) of biotin was 0.10 ± 0.03 nmol/min/mg protein, and the affinity (K_m_) was ca. 386.20 μM. Moreover, it was confirmed that biotin interacted with and utilized SMVT for its cellular uptake, since in the presence of the SMVT-utilizing inhibitors/competing substrates mixture (ketoprofen, pantothenic acid, and prostaglandin E_2_), the uptake was reduced (34.95% at 100 μM and by 38.05% at 150 μM biotin concentrations). Similar partial inhibition of biotin uptake has previously been reported in primary brain microvessel endothelial cells.^31^ Thus, in conclusion, biotin was taken up by U-87MG cells effectively, and the cellular uptake was proved to be SMVT-mediated.

**Figure 4:**
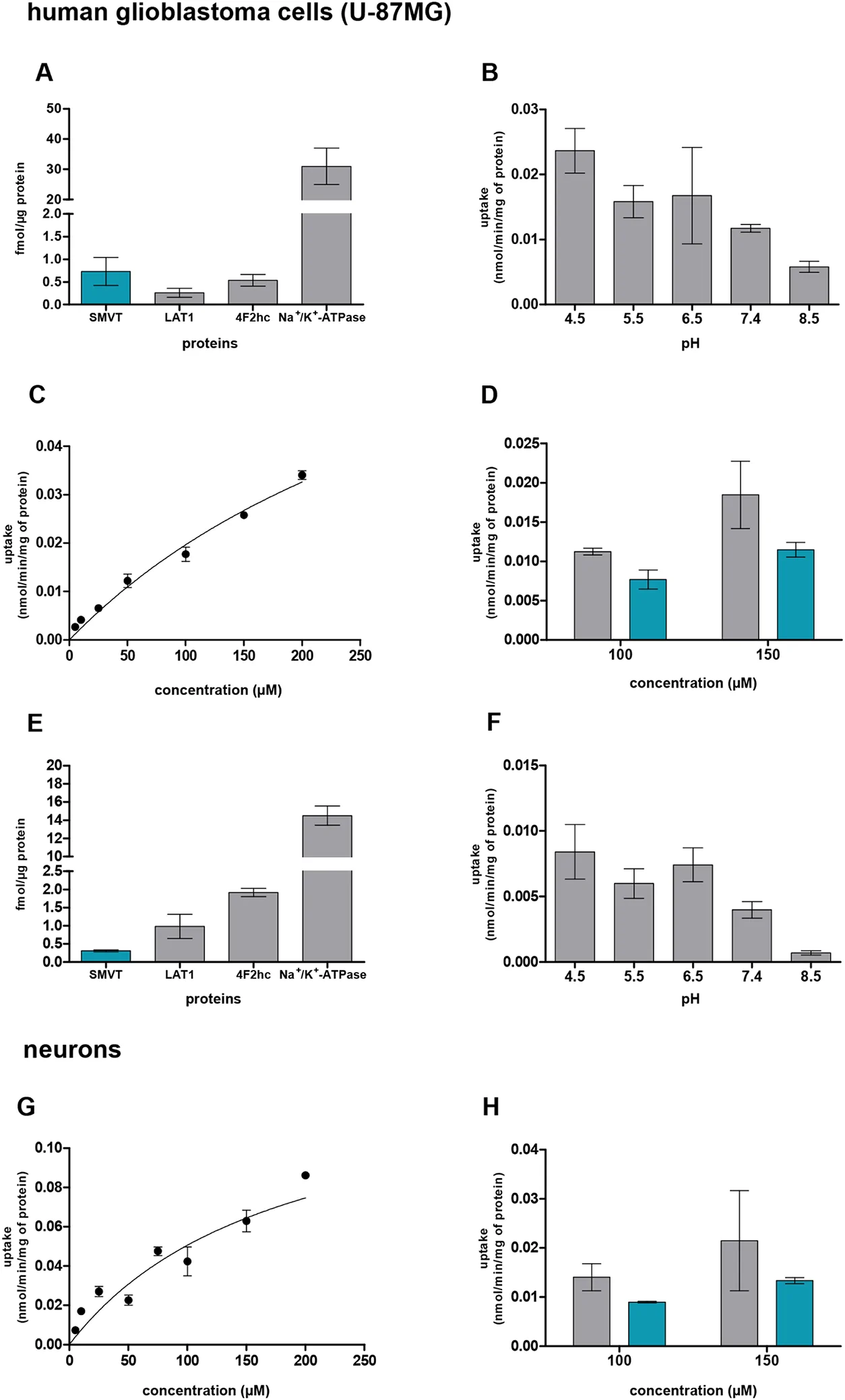
Characterization of SMVT in glioblastoma U-87MG cells (**A**) and primary mouse neurons (**E**): quantitative protein levels of sodium-dependent multivitamin transporter (SMVT), light and heavy subunits of the l-type amino acid transporter 1 (LAT1 and 4F2hc, respectively), and sodium–potassium adenosine triphosphatase (Na^+^/K^+^ ATPase) analyzed from the plasma membranes and normalized to the total amount of protein in the plasma membrane. The pH-dependent uptake (at pHs 4.5, 5.5, 6.5, 7.4, and 8.5) of biotin, cellular uptake of biotin (100–150 μM) in the presence (blue) or absence (gray) of an SMVT–inhibitor’s mixture (30 μM ketoprofen, 30 μM pantothenic acid, and 30 μM prostaglandin E_2_), and concentration-dependent uptake of 5–200 μM biotin (10 min incubation) in glioblastoma U-87MG cells (**B–D**) and primary mouse neurons (**F–H**). The results are presented as mean ± SD (*n* = 3, biological replicates). The inhibitory effect on cellular uptake was statistically nonsignificant, using two-way analysis of variance (ANOVA) followed by Bonferroni’s multiple comparisons test.

**Table 3:** Calculated Expression of Sodium-Dependent Multivitamin Transporter (SMVT), Michaelis–Menten Kinetic Parameters (*V*_max_ and K_m_), and Inhibition of Biotin Uptake (100 and 150 μM) in Human Glioblastoma Cells (U-87MG) and Mouse Neurons (Mean ± SD, n = 3)

	glioblastoma cells (U-87MG)	mouse neurons
Expression of SMVT (fmol/μg of protein)	0.73 ± 0.31	0.31 ± 0.03
Biotin transport capacity, *V*_max_ (nmol/min/mg protein)	0.10 ± 0.03	0.14 ± 0.04
Biotin affinity, K_m_ (μM)	386.20	182.00
Biotin uptake inhibition % (100 μM)	34.95	36.11
Biotin uptake inhibition % (150 μM)	38.05	37.93

Glioblastoma cells U-87MG provided a practical screening model for the early-stage evaluation and validation of the uptake studies. However, they differ biologically from primary mouse neurons. Therefore, SMVT expression and function were subsequently evaluated in primary mouse neurons. SMVT was found to be expressed in isolated mouse neurons at 0.31 ± 0.03 fmol/μg of protein (Figure 4E), confirming the presence of the transporter in the target cell type. In contrast to U-87MG cells, SMVT was expressed 3-fold less than LAT1 (0.98 ± 0.33 fmol/μg of protein) and almost 47-fold less than the housekeeping protein Na^+^/K^+^ ATPase (14.50 ± 1.06 fmol/μg of protein). Following the same trend with U-87MG cells, biotin uptake gradually decreased with increasing pH and was mediated by SMVT, since biotin uptake was reduced by 36.11% at 100 μM and by 37.93% at 150 μM biotin concentrations in the presence of an SMVT-utilizing inhibitors/competing substrates mixture (Figure 4F–H). Although the expression of SMVT in neurons was lower compared to glioma cells, biotin was transported more efficiently across the cell membrane in neurons (*V*_max_ = 0.14 ± 0.04 nmol/min/mg protein) with a higher affinity for SMVT (K_m_ ∼ 182.00 μM) than in U-87MG cells.

### 2.5 Compound **1** Utilizes SMVT and Accumulates Effectively in Mouse Neurons

To screen the initial affinity of novel compounds toward SMVT and to identify the most promising candidate for neuronal uptake, U-87MG cells were used for concentration-dependent uptake studies prior to experiments in primary mouse neurons. After 10 min incubation of the compounds in U-87MG cells, the most promising uptake was seen with COMP **1** (*V*_max_ = 0.005 ± 0.001 nmol/min/mg protein, K_m_ ∼ 30.87 μM), as shown in Figure 5A–D and Table 4. This finding is consistent with the compound design predictions, as COMP **1** demonstrated the most favorable binding profile. In mouse neurons, COMP **1** was taken up to a similar extent (*V*_max_ = 0.004 ± 0.001 nmol/min/mg protein, K_m_ ∼ 5.86 μM, Figure 5G). Other compounds were not easily taken up by U-87MG cells, and since the sensitivities of their LC-MS/MS methods were also relatively low, only COMP **1** was selected for further investigations, consistent with the prior docking results.

**Table 4:** The Calculated Michaelis–Menten Kinetic Parameters (*V*_max_ and K_m_), and Inhibition of COMP **1** Uptake at a Given Concentration of SMVT/OATP Inhibitors in Human Glioblastoma Cells (U-87MG) and Mouse Neurons (Mean ± SD, n = 3)

	glioblastoma cells (U-87MG)	mouse neurons
COMP **1** transport capacity, *V*_max_ (nmol/min/mg protein)	5.20 × 10^–3^ ± 0.88 × 10^–3^	4.09 × 10^–3^ ± 0.64 × 10^–3^
COMP **1** affinity, K_m_ (μM)	30.87	5.86
COMP **1** SMVT-mediated uptake inhibition % (50 μM)	68.19	-
COMP **1** OATP-mediated uptake inhibition % (100 μM)	90.07	-

Curiously, at higher concentrations of all derivatives (>100 μM), a sudden rise was observed, indicating possible utilization of another transporter. Based on the selected promoiety PAH, the organic anion transporter polypeptide (OATP) family was suspected. This hypothesis was confirmed by the addition of SMVT and OATP inhibitors to the incubation mixtures with U-87MG cells (Figure 5E-F). In the presence of SMVT inhibitors/competing substrates, only the uptake of 50 μM COMP **1** was reduced by 68.2% while in higher concentration (100 μM), the decrease was negligible. In contrast, in the presence of OATP inhibitors/competing substrates (naringin, rifampicin, diclofenac, flufenamic acid), the uptake of COMP **1** dramatically decreased by 90.07% and only at the higher (100 μM) COMP **1** concentration. Therefore, COMP **1** (>100 μM) is most likely able to utilize a secondary transport mechanism within the OATP family. Moreover, the hypothesis of two different transport mechanisms was further supported by Eadie-Hofstee analysis in both cell types (Figure 5A,**G**), which showed nonlinear behavior for COMP **1**, suggesting that its uptake is not mediated by a single Michaelis–Menten component. Notably, as already observed in plasma and tissue matrices, COMP **1** did not release creatine *in vitro*. However, we further investigated the COMP **1**’s brain protein binding, pharmacokinetics, and interactions with CK to assess its role within cellular energy metabolism.

**Figure 5:**
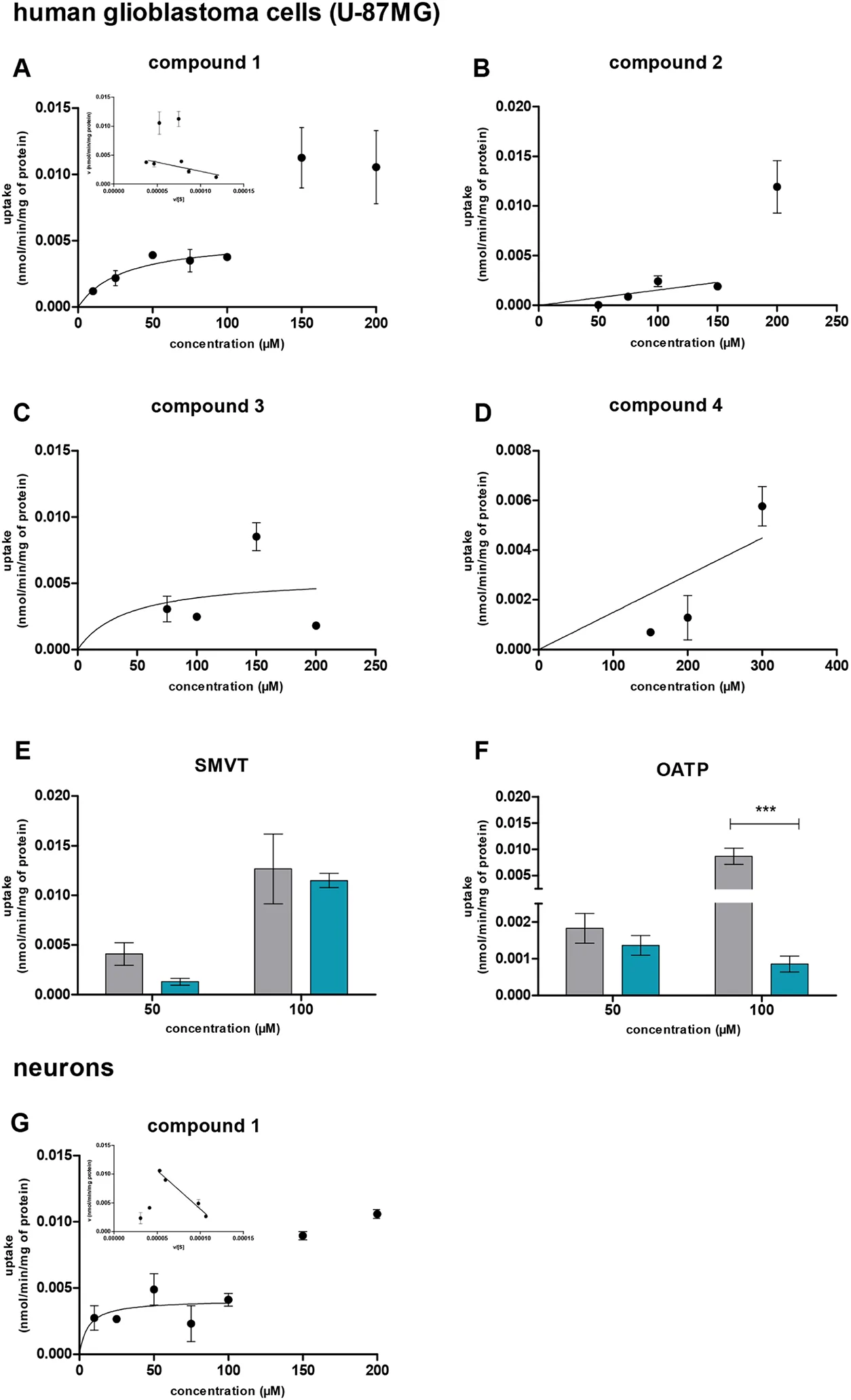
(**A–D**) The concentration-dependent cellular uptake of compound **1,** compound **2**, compound **3**, and compound **4** at several concentrations (10–200 μM, 10 min incubation). (**E–F**) The cellular uptake of compound **1** (50 and 100 μM) in the presence (blue) or absence (gray) of SMVT (pantothenic acid, ketoprofen, prostaglandin E_2_) and OATP (naringin, rifampicin, diclofenac, flufenamic acid) substrates in human glioblastoma U-87MG cells. (**G**) The most promising compound (COMP **1**, 10–200 μM, 10 min incubation) was taken up by mouse neurons. The Eadie-Hofstee plots for transporter-mediated uptake of COMP **1** are shown as insets. The results are presented as mean ± SD (*n* = 3, biological replicates). The inhibitory effect on cellular uptake was studied using two-way ANOVA followed by Bonferroni’s multiple comparisons test with a statistically significant difference ***P < 0.001.

### 2.6 Compound **1** Achieves a Relatively High Unbound Fraction in the Brain

As only the unbound drug can cross membranes, bind to receptors, and/or be pharmacologically active, the unspecific binding of COMP **1** in mouse brain S9 fraction, mouse serum, mouse liver S9 fraction, and mouse kidney S9 fraction was studied. As shown in Table 5, COMP **1** demonstrated a high unbound fraction (f_u_ = 66.21 ± 2.51%) in mouse serum, indicating low plasma protein binding and favorable conditions for BBB penetration. Furthermore, COMP **1** exhibited a high unbound fraction in kidney tissue (f_u_ = 62.94 ± 9.15%), whereas in liver tissue the unbound fraction was lower (f_u_, ca. 34.12%). Importantly, COMP **1** exhibited a reasonable unbound fraction in the brain (f_u_ = 31.99 ± 9.16%), suggesting favorable bioavailability and potential for effective CNS target engagement. To assess CNS exposure, the compound was further evaluated in a pharmacokinetic study.

**Table 5:** Unbound Fractions (f_u_) of Compound **1** in the Mouse Brain S9 Fraction, Mouse Serum, Mouse Liver S9 Fraction, and Mouse Kidney S9 Fraction^a^

	mouse brain S9	mouse serum	mouse liver S9	mouse kidney S9
Compound **1** (f_u_ as %)	31.99 ± 9.16	66.21 ± 2.51	ca. 34.12	62.94 ± 9.15

a Binding was determined *in vitro* using the equilibrium dialysis method The results are presented as mean SD (n = 3)

**Table 6:** The Calculated Area under the Curve (AUC) between 0 and 360 min, the Unbound Drug Partition in Tissue (AUC_u_), Peak Concentration (*C*_max_), Peak Time Point (*T*_max_), Elimination Phase Half-Life (*T*_1/2β_), the Distribution Coefficient (K_p_), and the Unbound Brain-To-Plasma Partition Coefficient (K_p,u_) of COMP **1** after a Bolus Injection (25 μmol/kg, I.P., n = 3 per Time Point)^a^

Tissue	AUC (nmol/mL × min, nmol/g × min)	AUC_u_ (mol/mL × min, nmol/g × min)	*C*_max_ (nmol/g, nmol/mL)	*T*_max_ (min)	*T*_1/2β_ (min)	K_p_ (AUC/AUC_plasma_)	K_p,u_ (AUC_u_/AUC_u,plasma_)
Plasma	197.40	130.69	26.69	5	4.45	-	-
Liver	21.56	*n.a.*	1.19	5	8.37	0.1092	*n.a.*
Brain	17.04	5.45	0.31	5	17.77	0.08632	0.0417
Kidney	24.25	15.26	2.48	5	5.44	0.1228	0.1168

a *n.a.* = not acquired because of missing data for calculation.

### 2.7 Creatine Compound **1** Accumulates in the Mouse Brain

COMP **1** (25 μmol/kg) was administered intraperitoneally (i.p.), and the distribution to different organs was evaluated between 0 and 360 min. The calculated *in vivo* pharmacokinetic parameters are presented in Table 6, and the time–concentration profiles of COMP **1** in plasma, liver, brain, and kidney are presented in Figure 6. Our data show that COMP **1** was rapidly absorbed into the systemic circulation after bolus i.p. administration and distributed to all tissues under investigation: brain, liver, and kidney. The peak concentration (*C*_max_) of COMP **1** was the highest in the liver (1.19 nmol/g), and the distribution was rapid to all tissues, as the time to reach the maximum plasma concentration (*T*_max_) was observed at the first measurement time point 5 min. To better understand the distribution kinetics between plasma and different organs, the distribution coefficients (K_p_ AUC ratios between tissue and plasma and K_p,u_, AUC ratios between unbound concentrations in tissue and plasma from Table 5) were calculated. Importantly, COMP **1** was distributed into the brain at a comparable level as in other tissues (K_p,brain_ = 0.09 vs K_p,liver_ = 0.11 and K_p,kidney_ = 0.12) and eliminated more slowly (half-life, *T*_1/2β_ = 17.77 min). Moreover, it exhibited AUC_u,brain_/AUC_u,plasma_ ratio of 0.04 which is in line with previously studied LAT1-utilizing conjugates.^32,33^ As shown in Figure 6C, after the initial distribution phase, the K_p,brain_ increased linearly over time. This behavior may indicate carrier-mediated uptake across the BBB; however, other kinetic processes may also contribute to the observed increase. In addition, based on the *in vitro* inhibition data, COMP **1** utilizes both SMVT and OATP depending on concentration; therefore, its brain accumulation may not be attributed exclusively to SMVT. Additional studies using *in vivo* transporter inhibition or transporter-deficient models will be required to confirm transporter-mediated transport across BBB and to define the relative contributions of SMVT and OATP transporters. Since several OATP subtypes are expressed in the brain, they may contribute to the brain distribution of COMP **1** and its overall pharmacokinetic profile. Further optimization and evaluation in relevant CTDS animal models will also be needed to determine whether therapeutically relevant brain levels of COMP **1** can be achieved. Nevertheless, since no released creatine was detected at any time point, consistent with the results gained from enzymatic bioconversion and cellular uptake studies, the ability of COMP **1** to interact with cellular energy metabolism was subsequently investigated.

**Figure 6:**
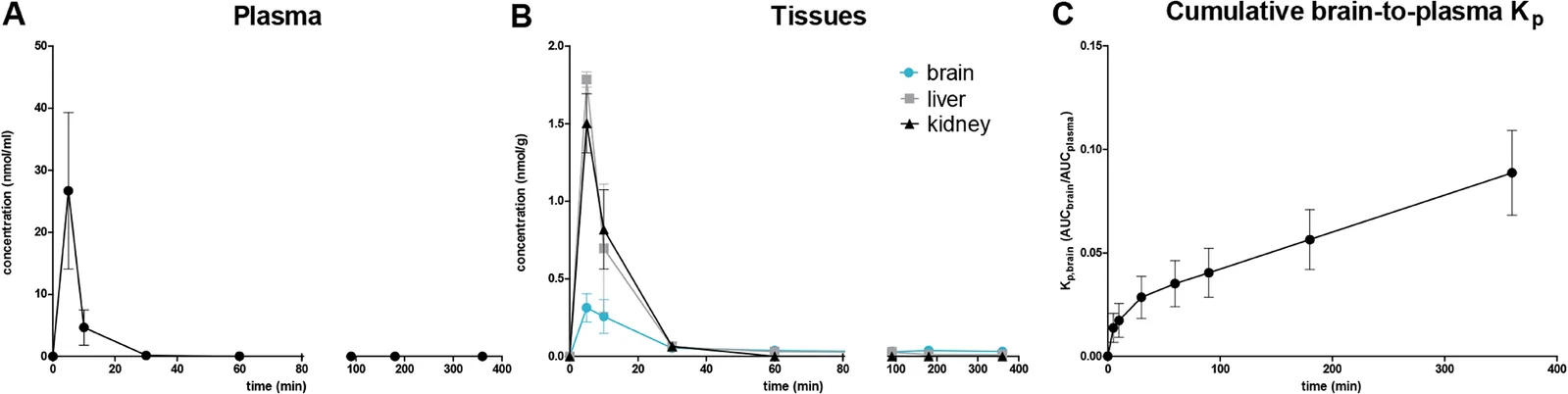
(A) The concentration–time curves between 0 and 360 min of COMP **1** in plasma, (**B**) tissues: brain, liver, and kidney after a single bolus dose (25 μmol/kg, i.p.), and (**C**) time-integrated brain-to-plasma ratios (K_p_) plot. Concentrations per time point are represented as mean ± SD (*n* = 3 for each time point).

### 2.8 Compound **1** Shows Creatine-Like Behavior in the Presence of CK

To evaluate whether COMP **1** can interact with the CK system, we first performed enzymatic assays measuring ATP and ADP concentrations over time. Although the compound did not undergo spontaneous hydrolysis to release free creatine, COMP **1** was evaluated for its potential interaction with CK and its possible participation in a creatine-like CK-catalyzed reaction according to Lohmann’s reaction:

$$\mathrm{Cr}+\mathrm{ATP}⇌\left(\mathrm{CK}\right)\mathrm{PCr}+\mathrm{ADP}+H^{+}$$

According to these results, ATP decreased time-dependently only when CK and COMP **1** were both present, whereas ATP remained stable in control over 1–60 min (Figure 7). An essentially identical profile was obtained with creatine: ATP decreased only with CK while remaining stable without CK. The most pronounced decrease in ATP concentration occurred at approximately 30 min, consistent with the peak enzymatic activity observed in similar *in vitro* studies.^34,35^ This was accompanied by a corresponding increase in ADP concentration, consistent with the reverse activity of CK under ATP-consuming conditions. Notably, an elevation of ADP was observed at the 30 min time point for both creatine and COMP **1**, suggesting comparable time-dependent behavior. Surprisingly, for COMP **1**, ADP levels also increased in control conditions lacking CK. This effect is likely related to limitations of the assay or to ATP hydrolysis or degradation through alternative nonspecific or enzymatic pathways outside CK-mediated catalysis, such as the activity of cytosolic ATPases or ectonucleotidases.^36^ Nevertheless, the decrease in ATP was significantly more prominent in the presence of CK, supporting the hypothesis that the novel derivative may serve as a functional substrate or cofactor in the CK-catalyzed reaction, without establishing kinetic equivalence to creatine. Importantly, in the absence of ATP in the assay buffer, ATP and ADP levels remained low and identical in CK and non-CK samples, confirming that COMP **1** does not generate or consume nucleotides in the absence of CK. To further investigate the reaction mechanism, we attempted to synthesize the phosphorylated derivative of COMP **1** expected to form during the CK reaction; however, the phosphorylation proved unexpectedly challenging, and the desired phosphorylated COMP **1** could not be obtained. Nevertheless, as additional, nonexperimental evidence, MD simulations were performed for both phosphorylated and nonphosphorylated COMP **1** to evaluate its potential interactions in a creatine-like context.

**Figure 7:**
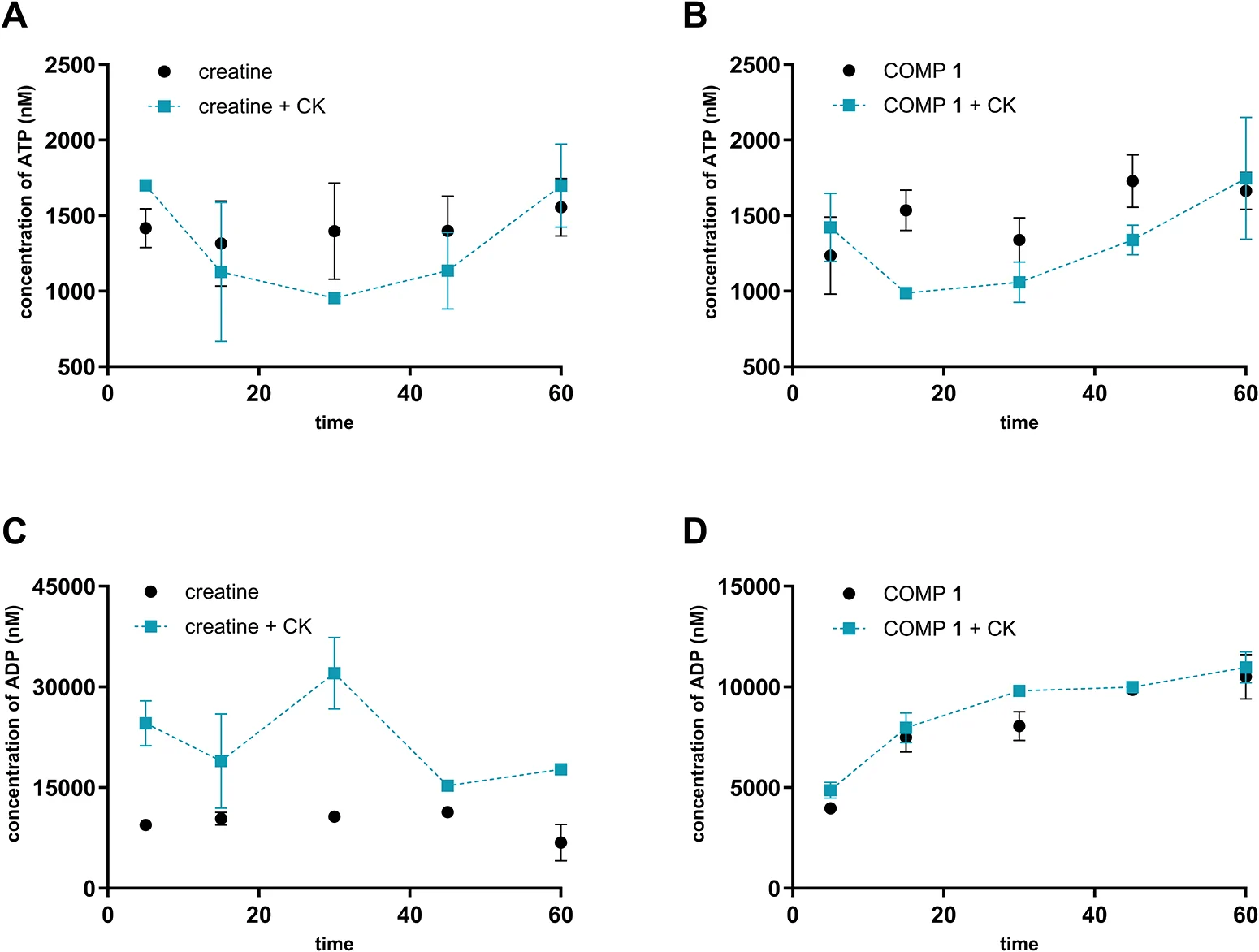
Time-dependent determination of adenosine triphosphate (ATP, **A-B**) and adenosine diphosphate (ADP, **C-D**) concentrations in different groups: (i) creatine, (ii) creatine with creatine kinase (CK), (iii) compound **1** (COMP **1**), and (iv) COMP **1** with CK. Samples were collected at defined time points and analyzed by LC-MS. The results are presented as mean ± SD (*n* = 3).

Next, examining COMP **1** interactions in the CK active site allowed an assessment of its substrate-like behavior. Analysis of binding poses, contact networks, and stability, in comparison to creatine, provided insight into the molecular basis of functional similarity.

Human ubiquitous mitochondrial creatine kinase (uMtCK; PDB: 1QK1) is the main isoform expressed in neurons, and therefore, it served as the basis for our computational studies.^37^ While all creatine kinase isoforms form dimers, mitochondrial isoforms, such as uMtCK, uniquely assemble into octamers.^38^ Each monomer contains an N-terminal domain (residues 1–95, six α-helices), a C-terminal domain (residues 120–379, eight-stranded β-sheet flanked by seven α-helices), and a connective linker (residues 96–119). The catalytic site, located in a deep cleft, binds creatine, ADP/ATP, and Mg^2+^, with the larger C-domain comprising most of the surrounding site and the N-domain capping the creatine-binding region.

We simulated five systems covering both nonphosphorylated and phosphorylated states: (i) COMP **1** with ATP, (ii) P-COMP **1** with ADP, (iii) creatine with ATP, (iv) P-creatine with ADP, and (v) ATP alone, together representing the distinct catalytic states. All systems included a coordinated Mg^2+^ ion to stabilize the complexes. The transition-state analogue geometry (Figure 8), i.e., with the nucleotide-coordinated Mg^2+^, was generated using the brain-type cytosolic creatine kinase (BBCK; PDB: 3B6R) as template.

**Figure 8:**
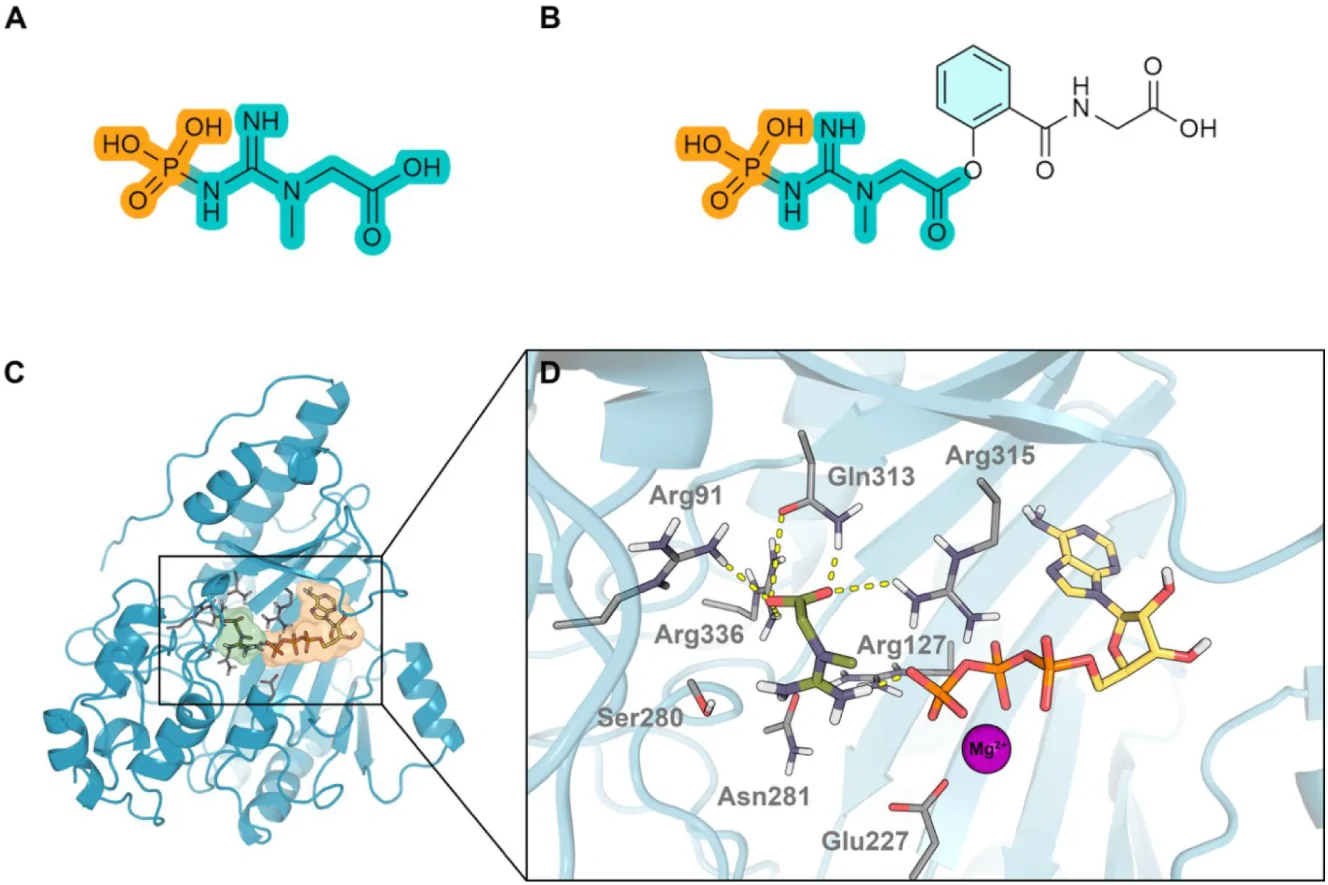
Structures of (**A**) phosphocreatine (P-creatine) and (**B**) phosphorylated COMP **1** (P-COMP **1**). (**C**) Overall structure of uMtCK with a zoomed view (**D**) of the catalytic site in the initial open state, populated by ATP (yellow-orange), Mg^2+^ (purple), and creatine (green). Creatine and ATP molecular surfaces are visualized in their respective colors.

MD simulations of substrate-bound complexes highlighted stability differences between phosphorylated and nonphosphorylated ligands. In the initial uMtCK model, the catalytic site was largely solvent-exposed (“open” state), resulting in poor retention of nonphosphorylated ligands. Creatine exited the binding site quickly (average residence time ∼10 ns), while COMP **1** remained bound in only three of five replicas. In contrast, phosphorylated ligands stabilized quickly through persistent electrostatic interactions between the phosphate group and Arg127 and Arg336 (Supporting Information Figures S7–S8), indicating that phosphorylation dominates binding stability in the open conformation. This instability of nonphosphorylated ligands is consistent with prior reports describing the open state of CK.^38^

To enable more meaningful comparisons of ligand-binding modes, a “closed” uMtCK conformation was identified and used for subsequent simulations. A 1000 ns MD simulation of ATP/Mg^2+^-bound uMtCK revealed a transition to a closed state, driven by the movements of loops His61–Lys65 and Gly316–Gly326 (Supporting Information Figure S9). Ligands were redocked into this closed model, and simulations were repeated using ADP/Mg^2+^ as the control system to better reflect a catalytically relevant environment.

In the closed binding site, COMP **1** displayed enhanced stability and a distinct, favorable binding mode relative to creatine. Although creatine eventually dissociated in all replicas, its average residence time increased significantly (145 ns versus 13 ns in the open state). COMP **1** initially replicated the canonical creatine interactions involved in phosphoryl transfer, with Glu227 orienting the guanidinium moiety toward the γ-phosphate (Figure 9A).^39^ At later stages, however, COMP **1** adopted an alternative pose characterized by hydrophobic positioning of the aromatic ring near loops Asn58–His61 and Leu197–Ala200, extension of the guanidinium group toward the α-phosphate, and stabilization of the carboxylate via Mg^2+^ coordination (Figure 9B). This rearrangement was associated with improved binding stability, suggesting that COMP **1** favors a binding mode distinct from, yet compatible with, creatine recognition.

**Figure 9:**
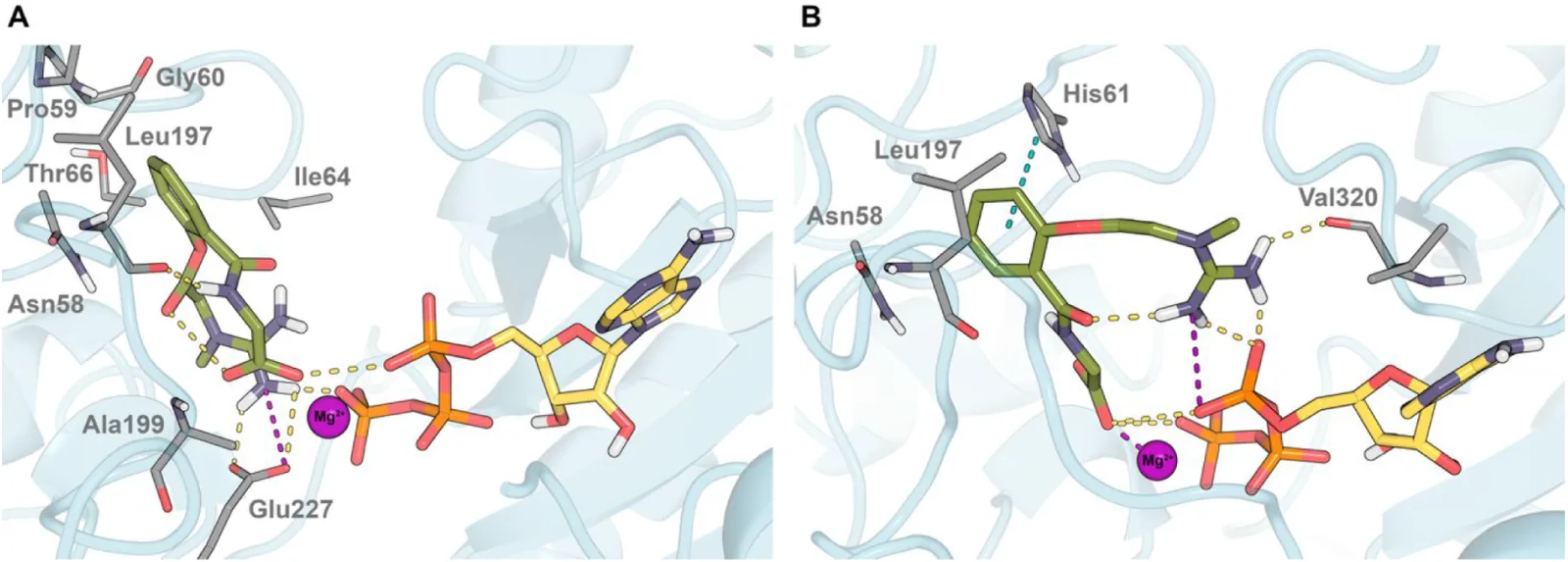
In the closed uMtCK conformation, (**A**) the guanidinium moiety in COMP **1** interacts with the γ-phosphate, coordinated by the Glu227 residue in the initial phase of the simulation. (**B**) Toward the end of the simulation, the guanidinium moiety in COMP **1** extends to interact with the α-phosphate, stabilized by the Val320 residue.

Phosphorylated COMP **1** and phosphorylated creatine behaved similarly in the closed state, but differed in their effects on protein dynamics. Both ligands engaged a common set of residues (Val67, Gly68, Glu227, Ser280, and Arg287) and were tightly anchored by Mg^2+^ coordination (Supporting Information Figure S10). However, P-creatine induced slightly higher protein RMSD and increased fluctuations in regions near the nucleotide-binding site (residues 186–189 and 316–324; Supporting Information Figures S11–S12), including a loop previously reported as disordered.^38^ These regions did not directly contact the ligands, suggesting longer-range dynamic effects specific to P-creatine. In contrast, P-COMP **1** produced a more stable dynamic profile. Taken together, these computational analyses suggest that COMP **1** engages CK through a distinct but functionally competent interaction mode relative to creatine, providing a mechanistic framework that complements the experimental data and could be extended by future targeted mutagenesis studies. Collectively, these findings further indicate that, although the novel derivative did not function as a prodrug and release creatine, it can interact with components of the CK system. However, further studies are required to determine whether this interaction has functional relevance for cellular energy metabolism in CTDS-relevant systems.

## 3 Methods

### 3.1 Molecular Modeling

#### 3.1.1 Model Preparation

The SMVT protein model was acquired from the AlphaFold database (Entry: Q9Y289, version 4, https://alphafold.ebi.ac.uk). The terminal tails (Met1–Thr23 and Arg551–Leu635), with low model confidence, were truncated. The model was prepared with the Protein Preparation Wizard,^40^ using default options except for capping the termini and minimizing the adjusted hydrogens. To estimate the most probable protonation, tautomeric, and ionization states, the physiological pH of 7.4 was used. The overall quality of the resulting structure was validated by the Ramachandran plot. Four outliers (Thr503, Thr505, Thr506, and Leu507) were identified in an extracellular loop located far from the binding site. Although this region exhibited low model confidence in the initial model, it was retained to preserve a continuous protein sequence, as its distance from the binding site suggests a negligible influence on ligand binding.

For the uMtCK protein model (PDB: 1QK1), chain A was used, with waters and the phosphate ion deleted. The homologous BBCK protein (PDB: 3B6R) was aligned with uMtCK. ADP, ATP, and Mg^2+^ were placed in the catalytic site of uMtCK based on their equivalent positions in BBCK. The complexes were prepared and validated following the procedures described for SMVT. Four outliers (Ala1, Asn93, Ile375, His379) were found, distal to the catalytic site. The disallowed angles were solved by performing local minimization. A similar procedure was followed for preparing the “closed” uMtCK conformation from the simulations. One outlier (Asp190) remained near the bound ATP.

#### 3.1.2 Molecular Docking

Compounds were either drawn in or imported to Maestro from the PubChem database (https://pubchem.ncbi.nlm.nih.gov/),^41^ and their structures were prepared using LigPrep.^42^ Default settings were used to predict all the relevant three-dimensional conformers at pH 7.4 ± 2.0 with the OPLS4 force field.

To predict the possible binding sites in the SMVT model, SiteMap was used with the default settings.^43^ The top-scoring site is in the central pore region, aligning well with the substrate-binding site in the homologous protein SMCT1 (PDB: 7SL9).^44^ This site was therefore used to dock the selected compounds. For SMVT, the receptor grid for molecular docking with Glide was generated using default settings.^45^ To position the grid center, the centroids of residues Val88 and Leu270 were used. For initial screening and refining the later-developed compound designs, extra-precision (XP) docking and flexible ligand sampling were used with default settings. The Induced Fit Docking protocol was employed to resolve issues with docking large compounds,^46^ allowing the receptor residues to adjust to the compounds. All resulting docking poses were then evaluated on the basis of the interaction terms and alignment with the natural substrates. The most promising poses were selected for study in MD simulations. For the uMtCK protein model, the procedure described for SMVT was followed. The receptor grid center was defined based on the equivalent position of creatine in BBCK. The Glide docking protocol with the XP scoring function was employed.

#### 3.1.3 Molecular Dynamics Simulations

MD simulations were carried out using Desmond.^47^ The system was created and prepared for simulations using the System Builder in Maestro. The SMVT protein was embedded in a POPC lipid bilayer. The membrane was placed using the predicted membrane orientation from the OPM database for the homologous protein SMCT1 (https://opm.phar.umich.edu/proteins/9479).^48^ The system was placed in an orthorhombic box with a 10 Å distance buffer from the solvent to the periodic box boundaries on each axis. The box was filled with TIP3P water.^49^ The total charge of the system was neutralized by adding either sodium or chloride ions. The OPLS4 force field was selected to be used for the simulations.^50^ The uMtCK systems were built using the same procedure described for SMVT, excluding membrane embedding.

All systems were simulated with five replicas, each configured with different random seeds to randomize the initial atomic velocities. After the default minimization, equilibration, and relaxation steps, the systems were simulated without positional restraints for 1000 ns under the NPT ensemble at 300 K and 1.01325 bar. The default time steps and ensemble settings were used.

Simulation trajectories were visually inspected for overall system stability, conformational changes, and ligand interactions with the binding site using PyMOL 3.0.0.^51^ The Simulation Interactions Diagram analysis in Maestro was run on the individual and combined system-specific trajectories from the replicas to calculate ligand-residue interaction fractions, as well as the RMSD for both the protein and the ligand. Protein backbone RMSD values over the simulation time were used to evaluate system equilibration. Selected trajectories were clustered using the trj_cluster.py script provided by Schrödinger. The centroid hierarchical clustering method was used with a 1.5 Å cutoff threshold, yielding the representative pose from the most populated cluster. The ligand, together with the surrounding pocket residues, was selected for RMSD calculation.

### 3.2 General Synthesis

All reactions were performed with commercial reagents obtained from Sigma-Aldrich (St. Louis, MO), BLD Pharmatech GmbH (Hamburg, Germany), Acros Organics (Waltham, MA), Thermo Fisher Scientific (Heysham, China), and Merck (Darmstadt, Germany). All solvents used in the reactions were anhydrous. Dichloromethane (DCM), *N,N*-dimethylformamide (DMF), and tetrahydrofuran (THF) were dried over molecular sieves (4 Å) and were stored under an inert atmosphere. All reactions were performed under an inert argon or nitrogen atmosphere unless otherwise specified. Selected reactions were performed in Microwave synthesizers (Biotage Initiator^+^) using either 10 or 20 mL glass reaction vials at appropriate temperatures. The reactions were monitored by thin-layer chromatography using aluminum sheets coated with silica gel 60 F245 (0.24 mm) with suitable visualization. Purifications by flash chromatography were performed on silica gel 60 (0.063–0.200 mm mesh). The ^1^H and ^13^C nuclear magnetic resonance (NMR) spectra were recorded on a Bruker Avance III HD Ascend 600 (^1^H, 600 MHz and ^13^C, 150 MHz) spectrometer. Tetramethylsilane (TMS) was used as an internal standard (IS) in NMR solvent only for deuterated chloroform; otherwise, the solvent peak was used as the standard. Chemical shifts (δ) are displayed in parts per million (ppm) and coupling constants (*J*) in hertz (Hz). The spectra were processed from the recorded FID files with MestReNova software (Mestrelab Research, S.L.U., Spain). The following abbreviations are used: s, singlet; d, doublet; dd, doublet of doublets; ddd, doublet of doublets of doublets; t, triplet; td, triplet of doublets; q, quartet; and m, multiplet. Mass spectrometry (MS) spectra were recorded by an Agilent 6410 Triple Quadrupole Mass Spectrometer (Agilent Technologies, Santa Clara, CA, USA) equipped with an electrospray ionization source (ESI). All compounds are >95% pure by HPLC and UHPLC analysis (see more details of instrumentation below) and by elemental analysis (EA; C, H, N) with a PerkinElmer 2400 Series II CHNS/O organic elemental analyzer (PerkinElmer Inc., Waltham, MA, USA). The HPLC/UHPLC chromatograms (Figures S13–S16) ^1^H and ^13^C NMRs (Figures S17, S18, S19, S20) of all final compounds **1–4** are provided in the Supporting Information.

#### 3.2.1 General Procedure A

Into a microwave-reactor glass vial (20 mL), the anhydrous creatine (1 equiv), 1-ethyl-3-(3-(dimethylamino)­propyl)­carbodiimide hydrochloride (EDCI·HCl; 1.2 equiv), and *N*,*N*-diisopropylethylamine (DIPEA; 3 equiv) were added at room temperature, and the solids were dissolved in dry DMF (10 mL). Under an inert atmosphere of argon, the reaction mixture was irradiated in a microwave reactor at 100 °C for 15 min, followed by the addition of acid (1 equiv). The resulting mixture was irradiated for the second time in a microwave reactor at 100 °C for 1 h 40 min. Upon completion, the reaction solution was concentrated to dryness, absorbed onto silica gel, and purified by flash column chromatography using 0 to 100% MeOH in DCM + 0.5% (v/v) of Et_3_N as the eluent.

#### 3.2.2 General Procedure B

Into a microwave reactor glass vial (20 mL), 4-hydroxyhippuric acid (1 equiv), EDCI·HCl (1.5 equiv), and DIPEA (3 equiv) were added at room temperature, and the solids were dissolved in dry DMF (10 mL). Under an inert atmosphere of argon, the reaction mixture was irradiated in a microwave reactor at 100 °C for 30 min, followed by the addition of anhydrous creatine (1 equiv). The resulting mixture was irradiated for the second time in a microwave reactor at 100 °C for 2 h. Upon completion, the reaction solution was concentrated to dryness, absorbed onto silica gel, and purified by flash column chromatography using 0 to 100% MeOH in DCM + 0.5% (v/v) of Et_3_N as the eluent.

*(2-((N-Carbamimidoyl-N-methylglycyl)­oxy)­benzoyl)­glycine* (COMP **1**). Following general procedure **A**, 2-hydroxyhippuric acid (1.14 mmol, 1 equiv) was added to the coupling reaction. After CombiFlash purification, the product (COMP **1**) was obtained as a white crystalline solid (115 mg, 0.37 mmol, 33%). ^**1**^ **H NMR** (600 MHz, DMSO-*d*_6_) 9.11 (t, *J* = 5.7 Hz, 1H), 7.87 (dd, *J* = 7.9, 1.7 Hz, 1H), 7.44–7.33 (m, 1H), 6.95–6.85 (m, 2H), 3.97 (d, *J* = 5.6 Hz, 2H), 3.69 (s, 2H), 2.91 (s, 3H). ^**13**^ **C NMR** (151 MHz, DMSO-*d*_6_) δ 180.00, 171.03, 170.75, 168.62, 159.50, 133.81, 128.23, 118.80, 117.32, 115.35, 55.96, 41.12, 30.72. Calculated **ESI-MS** for C_13_H_16_N_4_O_5_ 308.112, found [M–H_2_O]^+^ 291.000 and [M–H_2_O]^−^ 289.000. HPLC purity of COMP **1** (96.43%).

*(4-((N-Carbamimidoyl-N-methylglycyl)­oxy)­benzoyl)­glycine* (COMP **2**). Following general procedure **A**, 4-hydroxyhippuric acid (1.14 mmol, 1 equiv) was added to the coupling reaction. Then, the crude was purified by CombiFlash to obtain COMP **2** as a yellowish crystalline solid (76 mg, 22%). ^**1**^ **H NMR** (600 MHz, DMSO-*d*_6_) δ 11.04 (s, 1H), 9.95 (s, 1H), 8.37 (t, *J* = 5.9 Hz, 1H), 7.73 (d, *J* = 8.7 Hz, 2H), 6.80 (d, *J* = 8.7 Hz, 2H), 4.03 (s, 2H), 3.96 (d, *J* = 5.9 Hz, 2H), 2.97 (s, 3H). ^**13**^ **C NMR** (151 MHz, DMSO-*d*_6_) δ 179.83, 171.74, 165.98, 160.06, 157.53, 129.09 (2C), 125.08, 114.76 (2C), 51.13, 45.95, 30.39. Calculated **ESI-MS** for C_13_H_16_N_4_O_5_ 308.112, found [M–H_2_O]^+^ 291.000. HPLC purity of COMP **2** (95.02%).

*(4-(2-(1-Methylguanidino)­acetamido)­benzoyl)­glycine* (COMP **3**). Following general procedure **A**, *para*-aminohippuric acid (1.02 mmol, 1 equiv) was added to the coupling reaction. Then, the crude was purified by CombiFlash chromatography to obtain COMP **3** as a pale white crystalline solid (115 mg, 33%). ^**1**^ **H NMR** (600 MHz, DMSO-*d*_6_) δ 11.04 (s, 1H), 8.13 (t, *J* = 5.9 Hz, 1H), 7.58 (d, *J* = 8.6 Hz, 2H), 6.54 (d, *J* = 8.6 Hz, 2H), 5.59 (s, 2H), 4.03 (s, 2H), 3.94 (d, *J* = 5.8 Hz, 2H), 2.96 (s, 3H). ^**13**^ **C NMR** (151 MHz, DMSO-*d*_6_) δ 180.10, 171.74, 166.28, 157.45, 151.59, 128.70 (2C), 121.06, 112.53 (2C), 51.13, 45.90, 30.38. Calculated **ESI-MS** for C_13_H_17_N_5_O_4_ 307.128, found [M–H_2_O]^+^ 290.100 and [M–H_2_O]^−^ 288.000. HPLC purity of COMP **3** (99.72%).

*N-(N-((4-Hydroxybenzoyl)­glycyl)­carbamimidoyl)-N-methylglycine* (COMP **4**). Following the general procedure **B**, anhydrous creatine (1.02 mmol, 1 equiv) was added to the coupling reaction. Purification was done by CombiFlash chromatography to obtain COMP **4** as a pale yellow crystalline solid (50 mg, 16%). ^**1**^ **H NMR** (600 MHz, DMSO-*d*_6_) δ 11.04 (s, 1H), 9.95 (s, 1H), 8.37 (t, *J* = 5.9 Hz, 1H), 7.73 (d, *J* = 8.7 Hz, 2H), 6.80 (d, *J* = 8.6 Hz, 2H), 4.03 (s, 2H), 3.96 (d, *J* = 5.8 Hz, 2H), 3.64 (s, 1H), 2.97 (s, 3H). ^**13**^ **C NMR** (151 MHz, DMSO-*d*_6_) δ 179.83, 171.75, 165.99, 160.07, 157.54, 129.09 (2C), 125.08, 114.77 (2C), 51.14, 45.95, 30.40. Calculated **ESI-MS** for C_13_H_16_N_4_O_5_ 308.112, found [M–H_2_O]^+^ 291.000. HPLC purity of COMP **4** (96.96%).

### 3.3 General Bioanalytics

All reagents and solvents used in analytical studies were commercially available and of high purity, either analytical grade or ultragrade HPLC, purchased from Sigma (Sigma-Aldrich, MO, USA), J.T. Baker (Deventer, The Netherlands), Acros Organics (Waltham, MA, USA), AA Blocks (San Diego, CA, USA), VWR Chemicals (Radnor, PA, USA), BioLegend (San Diego, CA, USA), Abcam (Cambridge, UK), or Merck (Darmstadt, Germany). Water was purified using a Milli-Q (MQ) gradient system (Millipore, Milford, MA, USA). Creatine and P-creatine were purchased from Tokyo Chemical Industry Co., Ltd. (TCI) (Tokyo, Japan) and AK Scientific, Inc. (Union City, CA, USA), respectively.

The S9 fraction of mouse liver, kidney, and brain was prepared by homogenizing the tissues in 50 mM Tris-buffered saline (TBS) in a ratio of 1:4 (w/v) and centrifuging at 9000 × *g* for 20 min at 4 °C. The mouse brain cell homogenate (astrocytes and microglial cells) was prepared via sonication in TBS (0.5–1.0 × 10^6^ cells/ml). Pooled mouse serum was purchased from Biowest (Nuaillé, France), and pooled human plasma was obtained from healthy human donors (Finnish Red Cross, Helsinki). All biological samples were stored at −80 °C until further analysis.

Human glioblastoma cells (U-87MG, passages 10–25) were purchased from Lonza (Basel, Switzerland) and maintained in Dulbecco’s modified eagle medium (DMEM) containing 10% fetal bovine serum (FBS), 2 mM l-glutamine, 50 U/ml penicillin, and 50 μg/mL streptomycin at 37 °C, 5% CO_2_ incubator. In the uptake experiments, U-87MG cells were seeded at a density of 100,000 cells/well onto 24-well plates 1 day before the experiment. All studies were performed in three biological replicates from the same cell passage.

The mouse neurons were isolated from neonatal P0–2 pups of wild-type mice (C57BL/6JOlaHsd) as previously described,^52^ and maintained in Neurobasal medium supplemented with B-27, 2 mM l-glutamine, and 100 μg/mL primocin at 37 °C, 5% CO_2_ incubator. Cells were plated on poly-l-lysine-coated 24-well plates at a density of 50,000 cells per well, 7 days before the experiments. All experiments were performed using three biological replicates from the same cell passage. The mouse primary cell harvest from neonatal pups was conducted in compliance with the European Commission Directive 2010/63/EU and approved by the Institutional Animal Care and Use Committee of the University of Eastern Finland (Animal Usage Plan numbers: EKS-008-2024).

### 3.4 Chemical and Enzymatic Stability of Compounds **1**–**4**

The chemical stability of COMP **1**–**4** was determined at 37 °C in 0.1 M NaOH and 50 mM Tris-HCl buffer pH 7.4. Compounds (100 μM; DMSO ≤2%) were incubated with the corresponding reagent at 37 °C and 500 rpm for 24 h (Tris-HCl buffer) and 1 h (NaOH). Samples were collected at predetermined time points, mixed with ice-cold ACN (Tri-HCl buffer) or ice-cold ACN + 3.7% formic acid (FA; NaOH) in a ratio of 1:1 (v/v), centrifuged, and the supernatants were analyzed by the HPLC method described below.

The enzymatic stability of COMP **1–4** was assessed in human plasma, mouse serum, mouse liver S9 fraction, and mouse brain cell homogenate (astrocytes and microglial cells), each with a final protein concentration of 1 mg/mL. Compounds (DMSO ≤2%) were incubated in 50 mM Tris-HCl buffer pH 7.4 at 37 °C at 500 rpm for 6 h, using initial compound concentrations of 100 μM (mouse liver S9 fraction) or 200 μM (other matrices). At predetermined time points, reactions were stopped with ice-cold ACN in a ratio of 1:3 (v/v), centrifuged, and the supernatants were analyzed by the HPLC method described below. The pseudo-first-order half-lives (t_1/2_) for the rates of bioconversion of the prodrugs were calculated from the slope of the linear portion of the plotted logarithm of the remaining prodrug concentration versus time.

### 3.5 Quantitative Expression of SMVT in U-87MG and Mouse Neurons

The expression of the selected transmembrane proteins, SMVT (*Slc5a6*), LAT1 (*Slc7a5*), 4F2hc (*Slc3a2*), and Na+/K+-transporting ATPase subunits 1–4 (Na+/K+-ATPase 1–4, *Atp1a1–4*), was quantified from the plasma membrane fractions extracted from U-87MG and primary mouse neurons (n = 3 per studied cell type). The plasma membrane fractions were isolated using a commercial Membrane Protein Extraction Kit (Abcam, Cambridge, MA). The total protein contents for plasma membrane fractions were quantified using the Pierce BCA Protein Assay Kit (Thermo Fisher Scientific, Inc., Waltham, MA, USA) with a Hidex Sense microplate reader (Hidex Oy, Turku, Finland). Samples (50 μg protein per sample) were prepared according to established protocols^53^ and quantified using dynamic multiple reaction monitoring (dMRM) on a triple quadrupole mass spectrometer (QQQ 6495, Agilent Technologies, Santa Clara, CA, USA) as previously described.^54^ The acquisition parameters for the peptides used for protein quantification are described in the Supporting Information (Table S1). Quantification was based on the ratio of light/heavy standards, expressed as fmol/μg protein. The data were analyzed using the Agilent MassHunter quantitative analysis software for QQQ (version B.07.00) and processed by using Skyline software (version 23.1).

### 3.6 Transporter-Mediated Uptake of Biotin and Compounds into U-87MG Cells and Primary Mouse Neurons

For the following experiments, U-87MG cells and primary mouse neurons were cultured and prepared as described above (Section 3.3), washed with HBSS (125 mM NaCl, 4.8 mM KCl, 1.2 mM MgSO_4_, 1.3 mM KH_2_PO_4_, 1.3 mM CaCl_2_ × 2 H_2_O, 5.6 mM glucose, and 25 mM HEPES), and preincubated at the indicated pH values (4.5–8.5) for 10 min at 37 °C. Concentration-dependent uptake of biotin (5–200 μM) and COMP **1–4** (10–200 μM) was assessed in prewarmed HBSS, followed by incubation for 10 min at room temperature (RT). After incubation, cells were washed with ice-cold HBSS, lysed with 0.1 M NaOH, and samples were stored at −20 °C prior to LC-MS/MS analysis. For LC-MS/MS analysis, lysates were diluted with ACN and IS (200 nM labetalol and 200 nM diclofenac) in a ratio of 1:3 (v/v), centrifuged, and analyzed by the LC-MS/MS method as described below. Compound concentrations were determined from calibration curves and normalized to protein content. BSA (Sigma-Aldrich, MO, USA) was used as a standard protein for protein concentration, and the absorbance of samples (calculated as the mean of 3 samples/plate) was measured at a 595 nm wavelength by a multiplate reader (EnVision, PerkinElmer, Inc., Waltham, MA, USA).

Competitive uptake studies were performed using mixtures of SMVT-utilizing inhibitors/competing substrates (pantothenic acid, prostaglandin E_2_ketoprofen) or OATP-utilizing inhibitors/competing substrates (naringin, rifampicin, diclofenac, flufenamic acid). Cells were preincubated with inhibitors (total concentration, 100 μM) prior to exposure to biotin or COMP **1** at selected concentrations. Competitive uptake in the inhibitor was evaluated as a comparison of cellular uptake of biotin with and without the presence of a competitive substrate.

### 3.7 Nonspecific Brain Binding of Compound **1**

Nonspecific binding of creatine and its derivative was assessed by determining the unbound fractions (f_u_) in the mouse brain S9 fraction, mouse liver S9 fraction, mouse kidney S9 fraction, and mouse serum, using rapid equilibrium dialysis (RED) plates (Thermo Fisher Scientific, Inc., Waltham, MA, USA) and according to a previously described method.^55^ Compounds (20 μM) were incubated with the respective S9 fractions at 37 °C, samples were analyzed by LC-MS/MS, and the unbound fractions (f_u_) were calculated according to the previously published model.^56^

### 3.8 Pharmacokinetic Studies

The pharmacokinetic studies were performed with seven-week-old male mice (JAXC57BL/6J; n = 21; body weight: 25 ± 4 g) supplied by the Laboratory Animal Center of the University of Eastern Finland (Kuopio, Finland). Mice were housed under the following conditions: an ambient temperature of 22 ± 2 °C, a relative humidity of 55 ± 15%, a 12/12 h light–dark cycle, and tap water and food pellets provided *ad libitum* (Teklad 2016, Envigo, Netherlands). All experiments were performed during the light phase. All procedures were conducted under a license (ESAVI-2020-025070) approved by the Finnish National Animal Experiment Board and in accordance with the European Community Guidelines (Directive 2010/63/EU) and “Principles of Laboratory Animal Care” (NIH publication #85-23, revised in 1985). All options were considered to minimize the suffering and the number of animals.

Stock solutions (80 mM) of the studied compounds were prepared in DMSO 1 day before the study and were dosed to animals as a solution in saline containing 30% (w/v) Macrogol (15)-hydroxystearate (BASF SE, Germany) to yield a 2.5 mM formulation with a final DMSO concentration of 3%. Mice were dosed via i.p. bolus administration (25 μmol/kg) and anesthetized using pentobarbital (120 mg/kg i.p., Euthoxin Vet, Chanelle Pharmaceuticals Manufacturing Ltd., Ireland) approximately 5 min before the transcardial perfusion of ice-cold physiological saline for 1 min. Blood and tissue samples were collected at selected time points after injection. Plasma was separated by vortexing blood samples with 5 μL of 1000 IU heparin, centrifuged at 1500 × *g* for 10 min at 4 °C, and the supernatant was collected. Tissue samples were snap-frozen in liquid nitrogen and stored at −80 °C.

The frozen tissue samples were homogenized in 50 mM Tris buffer (pH 7.4; according to tissue weight 1:4) using a bead mill homogenizer (Omni Bead Ruptor 24 Elite homogenizer with a BR Cryo cooling unit, Omni International, Kennesaw, GA, USA). Subsequently, samples were diluted 1:3 (v/v) with ACN containing labetalol as the IS and 0.1% FA, centrifuged, and the supernatants were collected for LC-MS/MS analysis as described below.

### 3.9 High-Performance Liquid Chromatography (HPLC) Analyses of Studied Compounds

To determine the amounts of COMP **1**–**4**, creatine, and creatinine in stability and bioconversion studies, the HPLC system consisting of an Agilent 1100 binary pump (Agilent Technologies, Santa Clara, CA, USA) together with an HP 1050 variable-wavelength detector (for COMP **1**, **2**, and **4**) operated at 260–270 nm, and a UHPLC-UV system consisting of an Agilent 1290 Infinity II LC System (Agilent Technologies, Santa Clara, CA, USA) combined with an Agilent 1290 Infinity II Diode Array Detector (for COMP **3,** creatine, and creatinine) operated at wavelengths of 260 (COMP **3**) and 200 nm (creatine and creatinine) were used. For COMP **1** and **4**, the chromatographic separations were achieved on a ZORBAX SB-Phenyl column (4.6 × 150 mm, 5 μm; Agilent Technologies, Santa Clara, CA, USA) by using isocratic elution of MQ + 0.1% FA (A) and ACN + 0.1% FA (B) at a flow rate of 0.8 mL/min with a ratio of 80:20 (v/v). COMP **2** was separated on an Agilent ZORBAX SB-C18 analytical column (4.4 mm × 150 mm, 5 μm; Agilent Technologies, Santa Clara, CA, USA) by using isocratic elution of MQ + 0.1% FA (A) and ACN + 0.1% FA (B) at a flow rate of 0.8 mL/min with a ratio of 78:22 (v/v). For COMP **3**, creatine and creatinine, an ACQUITY UPLC BEH Amide column (1.7 μm, 2.1 mm × 100 mm; Waters Corporation, Milford, MA, USA) with MQ + 0.1% phosphoric acid (A) and 100% ACN (B) at a flow rate of 0.3 mL/min with ratios of 20:80 (COMP **3**) and 30:70 (creatine and creatinine) was used. Retention time for COMP **1** was 7.8 min, COMP **2** 3.2 min, COMP **3** 1.4 min, COMP **4** 3.5 min, creatine 2.2 min, and creatinine 1.5 min. The lower limit of quantification for COMP **1–4**, creatine, and creatinine was 0.05 μM. These HPLC methods were also accurate, precise, and selective over the range of 0.05–50 μM.

### 3.10 Liquid Chromatography–Mass Spectrometry (LC-MS) Analyses of Studied Compounds

The concentrations of biotin, COMP **1–4,** creatine, and P-creatine were quantified by an Agilent 1200 Series Rapid Resolution LC System coupled with an Agilent 6410 Triple Quadrupole Mass Spectrometer equipped with an electrospray ionization source (ESI-LC-MS/MS, Agilent Technologies, Santa Clara, CA, USA). The following conditions were applied: a drying gas (nitrogen) flow of 8 l/min with a temperature set at 300 °C, a nebulizer gas pressure of 40 psi, and a capillary voltage of 4 kV. LC-MS/MS data acquisition was performed in positive mode (ESI+, using labetalol as an IS), or in negative mode (ESI–, using diclofenac as an IS).

Briefly, biotin and the studied COMP **1–4** were separated by a reverse-phase column Zobrax Eclipse XDB-C18 (4.6 mm × 50 mm, 1.8 μm; Agilent Technologies, Santa Clara, CA, USA) set at 40 °C by using a mobile phase composed of MQ + 0.1% FA (A) and ACN + 0.1% FA (B) at a flow rate of 0.3 mL/min with a gradient: 0–1 min: 5% B, 1–3 min: 5% B → 95%, 3–6 min: 95% B, 6–6.5 min: 95% B → 5%, 6.5–8 min: 5% B. The sample injection volume was 5 μL. Analyte quantification was performed in positive mode (ESI+) using multiple reaction monitoring (MRM) with the transitions: 245 → 227 and 245 → 97 for biotin, 291 → 178, 291 → 150, and 291 → 141 for COMP **1**, 291 → 178, 291 → 150, and 291 → 114 for COMP **2**, 290 → 251, 290 → 177, and 290 → 114 for COMP **3**, 291 → 178, 291 → 150, and 291 → 114 for COMP **4,** and 329 → 294 and 329 → 162 for labetalol. The fragmentor voltages for biotin, COMP **1**, COMP **2**, COMP **3**, COMP **4**, and labetalol were 70 V, 100 V, 100 V, 100 V, 100 V, and 70 V, respectively, and the collision energies for product ions were 11 V, 14 V (biotin), 4 V, 4 V, 4 V (COMP **1**), 3 V, 5 V, 3 V (COMP **2**), 3 V, 3 V, 3 V (COMP **3**), 2 V, 5 V, 3 V (COMP **4**), and 10 V, 10 V (labetalol).

For creatine, creatinine, and P-creatine quantification, creatine and creatinine were separated by a Zobrax Eclipse XDB-C18 (4.6 mm × 50 mm, 1.8 μm; Agilent Technologies, Santa Clara, CA, USA) set at 40 °C by using a mobile phase composed of MQ + 0.1% FA (A) and ACN + 0.1% FA (B) at a flow rate of 0.25 mL/min with a gradient: 0–1 min: 5% B, 1–3 min: 5% B → 90%, 3–6 min: 90% B, 6–6.5 min: 90% B → 5%, 6.5–8 min: 5% B. The sample injection volume was 5 μL. LC-MS/MS data acquisition was performed in positive mode (ESI+) with the MRM transitions: 132 → 114, 132 → 90, and 132 → 86 for creatine, 114 → 86 and 114 → 44 for creatinine, and 329 → 294 and 329 → 162 for labetalol. The fragmentor voltages for creatine, creatinine, and labetalol were 50 and 100 V, and 70 V, respectively, and the collision energies were 10 V, 7 V, 9 V (creatine), 7 V, 10 V (creatinine), and 10 V, 10 V (labetalol). For P-creatine quantification, P-creatine was separated by a Supelco Discovery HS F5 (5 cm × 2.1 mm, 3 μm; Supelco Analytical, Bellefonte, PA, USA) set to 40 °C by using a mobile phase composed of MQ + 0.1% FA (A) and 100% ACN (B) with a flow rate of 0.2 mL/min and a gradient: 0–1 min: 0% B, 1–3 min: 0% B → 98%, 3–6 min: 98% B, 6–6.5 min: 98% B → 0%, 6.5–8 min: 0% B. The sample injection volume was 5 μL. LC-MS/MS data acquisition was performed in negative mode (ESI−) with diclofenac as an IS, and the transitions were 210 → 121, 210 → 97.1, and 210 → 79 for P-creatine, and 294 → 250 for diclofenac. The fragmentor voltages for P-creatine and diclofenac were 100 and 80 V, respectively, and collision energies were 5 V, 5 V, 15 V (P-creatine), and 2 V (diclofenac).

The data acquisition software was Agilent MassHunter Workstation software (version B.03.01), whereas the Quantitative Analysis (B.09.00) software was used for data processing and analysis. All methods were linear, accurate, selective, and precise (deviation ≤20% of the nominal concentrations) in a calibration range of 0.005–10 μM.

### 3.11 Creatine Kinase Assay

The reaction mixture with CK consisted of 2 mM ATP, 5 mM creatine or COMP **1**, 15 U/ml CK, and reaction buffer (50 mM Tris-HCl pH 7.5, 10 mM MgCl_2_, and 50 mM NaCl). The mixture without CK consisted of the same components except for CK. The mixtures (n = 3 per time point) were incubated at 37 °C at 500 rpm for 1 h, and at predetermined time points (5, 15, 30, 45, and 60 min), 50 μL of the reaction mixture was withdrawn from each sample. The enzymatic reaction was terminated by adding ice-cold ACN at a 1:3 ratio (v/v), incubated on ice for 10 min, diluted 1:100 (v/v) with 80% ACN containting ganciclovir as the IS, centrifuged at 14,000 × *g* for 10 min at 4 °C, and the supernatant transferred to HPLC vials.

ATP and ADP were quantified with an LC 1290 Infinity and an MS/MS 6495 (Agilent Technologies, CA, USA) with an electrospray ionization source (ESI-LC-MS/MS, Agilent Technologies, Santa Clara, CA, USA). ATP and ADP were separated by a Hypercarb Porous Graphitic Carbon analytical column (50 × 2.1 mm, 3 μm, 250 Å; Thermo Fisher Scientific, Waltham, MA, USA) set at 40 °C, and the mobile phase was composed of 1 mM ammonium fluoride in MQ (A) and 100% ACN (B). The mobile phase flow rate was 0.3 mL/min with a gradient: 0–0.5 min: 5% B, 0.5–5 min: 5% B → 80%, 5–7 min: 80% B, 7–7.1 min: 80% B → 5%, 7.1–10 min: 5% B. The sample injection volume was 5 μL. LC-MS/MS data acquisition was performed in positive mode (ESI+), using ganciclovir as an IS. The following conditions were applied: a drying gas (nitrogen) flow of 14 l/min with a temperature set at 200 °C, a nebulizer gas pressure of 40 psi, and a capillary voltage of 4 kV. Analyte quantification was performed using MRM with the transitions: 508 → 410 and 508 → 136 for ATP, 428 → 347.8 and 428 → 136 for ADP, and 256.2 → 152.1 and 256.2 → 134.9 for ganciclovir. The fragmentor voltages for ATP, ADP, and ganciclovir were 380 V, and the collision energies for product ions of ATP, ADP, and ganciclovir were 24 and 30 V (ATP), 16 and 16 V (ADP), and 21 and 25 V (ganciclovir). The data acquisition software was Agilent MassHunter Workstation software (version B.06.00), whereas Quantitative Analysis (B.09.00) software was used for data processing and analysis. The method was linear, accurate, selective, and precise (deviation ≤20% of the nominal concentrations) in a calibration range of 0.005–10 μM.

### 3.12 Statistical Analyses

All statistical analyses were performed using GraphPad Prism software (version 5.03, San Diego, CA, USA). Data are presented as mean ± SD, with statistically significant differences denoted by *P < 0.05, **P < 0.01, and ***P < 0.001. The inhibitory effect on cellular uptake was studied using a two-way ANOVA followed by Bonferroni’s multiple comparisons test.

## 4 Conclusions

This study highlights SMVT as a potential transporter involved in the brain and neuronal uptake of creatine derivatives as a relevant tool for further CTDS research. SMVT was functionally expressed in both human glioblastoma and mouse neuronal cells, with glioblastoma cells providing a practical screening model to facilitate early-stage evaluation prior to studies in primary neurons. *In silico* analyses identified PAH as a potential scaffold for SMVT recognition, which was further conjugated to creatinine. Among the four synthesized SMVT-targeting creatine derivatives, COMP **1**, where creatine was attached to the phenolic group of hippuric acid at the *ortho*-position, showed efficient SMVT-mediated uptake, neuronal accumulation, and the ability to cross the BBB. However, the brain concentrations were substantially lower than endogenous creatine levels, and the rapid decline in brain exposure suggests potential clearance mechanisms that may limit the efficacy. Compound **1** interacted with the CK system and retained key interactions within the kinase catalytic site according to MD simulations. Thus, further structural optimization will be needed to improve brain retention and enhance interactions with the CK system. Together, these findings highlight SMVT as a promising route for brain delivery of creatine analogues and support further investigation of SMVT-targeting compounds in relevant disease models. However, the extent to which these compounds can compensate for impaired energy metabolism remains to be established, for example, in CRT1-deficient *in vivo* models.

## Supplementary Material



## Data Availability

The raw MD trajectories, and associated protein–ligand interaction data are available in Zenodo repositories (SMVT: https://doi.org/10.5281/zenodo.15236194, uMtCK: https://doi.org/10.5281/zenodo.15236329).

## Notes

Figures S1–S5: SMVT interaction frequency analyses from MD simulations; Figure S6: Ligand RMSD analyses for SMVT complexes; Figures S7–S12: MD analyses of open and closed uMtCK complexes, including ligand RMSD, interaction frequencies, conformational changes, protein RMSD, and protein RMSF; Figures S13–S16: HPLC/UHPLC chromatograms and purity analyses of compounds 1–4; Figures S17–S20: 1H and 13C NMR spectra of compounds 1–4; Table S1: Transitions of the targeted LC-MS plasma membrane proteomics (PDF)

## Abbreviations

ADP: adenosine diphosphate
CAN: acetonitrile
ANOVA: analysis of variance
ATP: adenosine triphosphate
AUC: area under the curve
BBB: blood-brain barrier
Creatine: creatine
P-creatine: creatine-phosphate
Creatinine: creatinine
CRT1: creatine transporter 1
CK: creatine kinase
*C*_max_: peak concentration
CNS: central nervous system
COMP: 1–4, compound 1–4
CTDS: creatine transporter deficiency syndrome
DCM: dichloromethane
DMF: *N*,*N*-dimethylformamide
IS: internal standard
K_m_: Michaelis–Menten constant
K_p_: distribution coefficient
LAT1: l-type amino acid transporter 1
MD: molecular dynamics
Na^+^/K^+^ATPase: sodium–potassium adenosine triphosphatase
SMVT: sodium-dependent multivitamin transporter
THF: tetrahydrofuran
*t*_max_: peak time point
*V*_max_: maximum transport capacity.
