# Supplementary material for: Sodium-Dependent Multivitamin Transporter Facilitates Brain and Neuronal Uptake of a Creatine Analogue
Source: ACS Chem Neurosci. 2026 Sep 17;17(21):3545–61. doi: 10.1021/acschemneuro.6c00484 (PMC13741793; doi:10.1021/acschemneuro.6c00484)
Supplement: Supplementary file 1 [file cn6c00484_si_001.pdf]

Supporting Information

Sodium-Dependent Multivitamin Transporter Facilitates Brain and Neuronal Uptake of a Creatine Analogue

AUTHOR NAMES

Adéla Králová[1*,#], Ville Kuorikoski[1#], Landry Anamea[1], Janne Tampio[1], Arun Kumar Tonduru[1], Hengjing Cui[2], Henri Leinonen[1], Jaana Rysä[1], Antti Poso[1], Tetsuya Terasaki[1], Aaro Jalkanen[1], Thales Kronenberger[1,3], Kristiina M. Huttunen[1]

AUTHOR ADDRESS

[1]School of Pharmacy, Faculty of Health Sciences, University of Eastern Finland, 70211 Kuopio, Finland

[2]Department of Pharmacology, Faculty of Medicine, University of Helsinki, Helsinki 00290, Finland

[3]Interfaculty Institute of Microbiology and Infection Medicine (IMIT), University of Tübingen, and German Center for Infection Research (DZIF), Partner Site Tübingen, Elfriede-Aulhorn-Str. 6, Tübingen 72076, Germany

# These authors contributed equally

*Corresponding author: University of Eastern Finland, P.O. Box 1627, 70211 Kuopio, Finland, E-mail: adela.kralova@uef.fi

Figure S1. (A–F) Interaction frequencies for SMVT complexes bound with selected screening compounds.............4

Figure S2. (A–F) Ligand carboxyl group interaction frequencies for SMVT complexes bound with selected screening compounds 5

Figure S3. (A–D) Ligand carboxyl group interaction frequencies for SMVT complexes bound with COMP **1–4**. 6

Figure S4. (A–D) Interaction frequencies for SMVT complexes bound with COMP **1–4** 7

Figure S5. Combined carboxyl and guanidine group interaction frequencies for SMVT complexed with COMP **1**. ..8

Figure S6. (A–D) The Ligand Root Mean Square Deviations (L-RMSD) for SMVT complexes bound with COMP **1–4**..........9

Figure S7. (A–D) The Ligand Root Mean Square Deviations (L-RMSD) for "open" uMtCK complexes bound with phosphorylated and non-phosphorylated COMP **1** and creatine 10

Figure S8. (A–C) Interaction frequencies for "open" uMtCK complexes bound with phosphorylated and non-phosphorylated COMP **1** and creatine 11

Figure S9. View of the surrounding loops near the uMtCK catalytic site, visualizing the change in loop distance during the transition from "open" (cyan) to "closed" (orange) state 12

Figure S10. (A–C) Interaction frequencies for "closed" uMtCK complexes bound with phosphorylated and non-phosphorylated COMP **1** and creatine 13

Figure S11. (A–E) The Protein Root Mean Square Deviations (P-RMSD) for "closed" uMtCK apo (ADP) and bound phosphorylated and non-phosphorylated COMP **1** and creatine complexes 14

Figure S12. (A–E) The Protein Root Mean Square Fluctuations (P-RMSF) for "closed" uMtCK apo (ADP) and bound phosphorylated and non-phosphorylated COMP **1** and creatine complexes 15

Figure S13. HPLC chromatogram of compound **1** (purity 96.43%)..........16

Figure S14. HPLC chromatogram of compound **2** (purity 95.02%)..........16

Figure S15. UHPLC chromatogram of compound **3** (purity 99.72%)..........17

Figure S16. HPLC chromatogram of compound **4** (purity 96.96%)..........17

Figure S17. ^{1}H and ^{13}C NMR spectra of compound **1**. 19

Figure S18. ^{1}H and ^{13}C NMR spectra of compound **2**. 19

Figure S19. ^{1}H and ^{13}C NMR spectra of compound **3**. 20

Figure S20. ^{1}H and ^{13}C NMR spectra of compound **4**. 21

Table S1. Transitions of the targeted LC-MS plasma membrane proteomics. 22

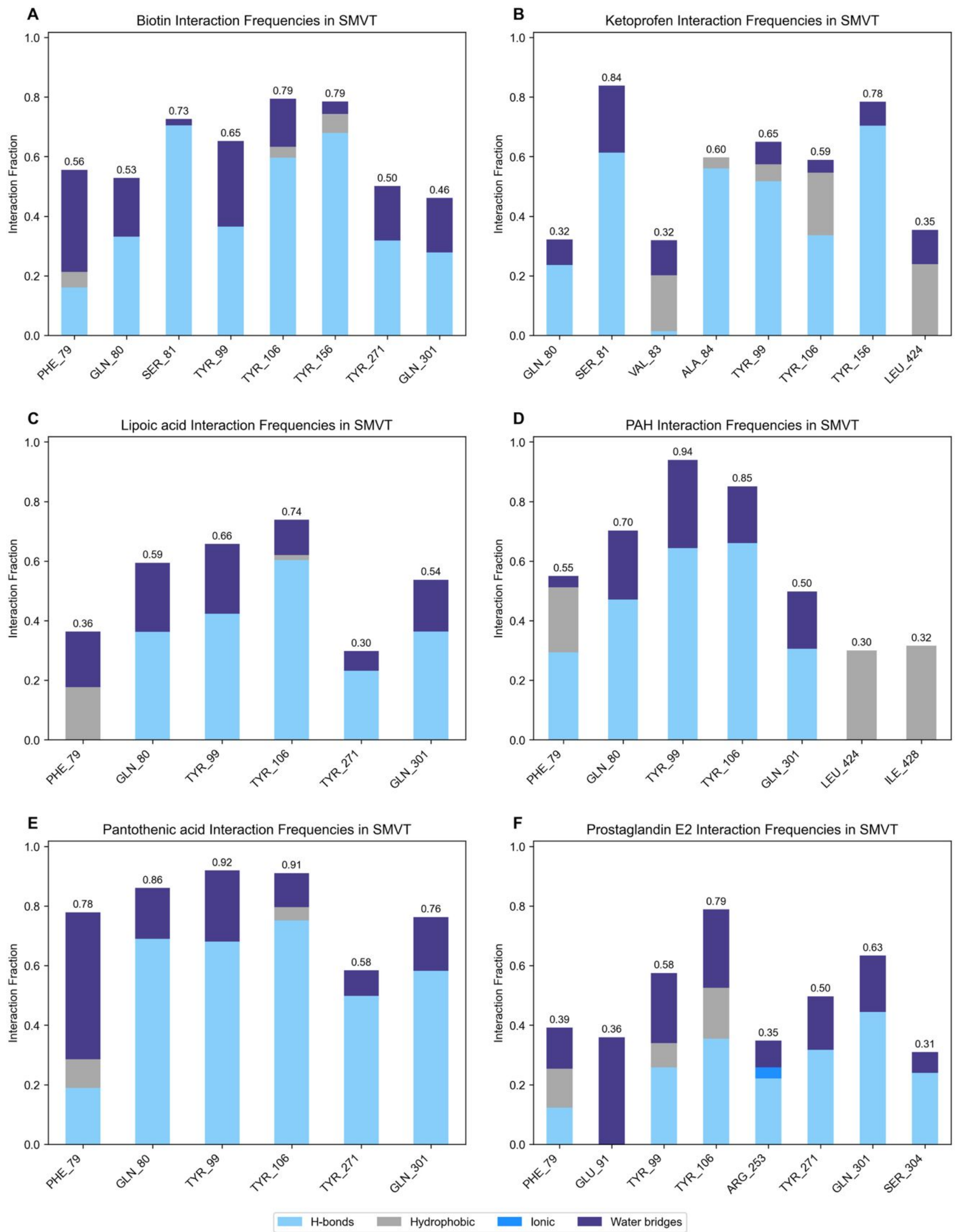
A
Biotin Interaction Frequencies in SMVT
Interaction Fraction
0.56 0.53 0.73 0.65 0.79 0.79 0.50 0.46
PHE_79 GLN_80 SER_81 TYR_99 TYR_106 TYR_156 TYR_271 GLN_301
B
Ketoprofen Interaction Frequencies in SMVT
Interaction Fraction
0.32 0.84 0.32 0.60 0.65 0.59 0.78 0.35
GLN_80 SER_81 VAL_83 ALA_84 TYR_99 TYR_106 TYR_156 LEU_424
C
Lipoic acid Interaction Frequencies in SMVT
Interaction Fraction
0.36 0.59 0.66 0.74 0.30 0.54
PHE_79 GLN_80 TYR_99 TYR_106 TYR_271 GLN_301
D
PAH Interaction Frequencies in SMVT
Interaction Fraction
0.55 0.70 0.94 0.85 0.50 0.30 0.32
PHE_79 GLN_80 TYR_99 TYR_106 GLN_301 LEU_424 ILE_428
E
Pantothenic acid Interaction Frequencies in SMVT
Interaction Fraction
0.78 0.86 0.92 0.91 0.58 0.76
PHE_79 GLN_80 TYR_99 TYR_106 TYR_271 GLN_301
F
Prostaglandin E2 Interaction Frequencies in SMVT
Interaction Fraction
0.39 0.36 0.58 0.79 0.35 0.50 0.63 0.31
PHE_79 GLU_91 TYR_99 TYR_106 ARG_253 TYR_271 GLN_301 SER_304
H-bonds
Hydrophobic
Ionic
Water bridges

Figure S1. (A–F) Interaction frequencies for SMVT complexes bound with selected screening compounds. Interactions shown have a frequency > 0.3, based on merged data from the five replicas where the ligand remained stable in the binding site.

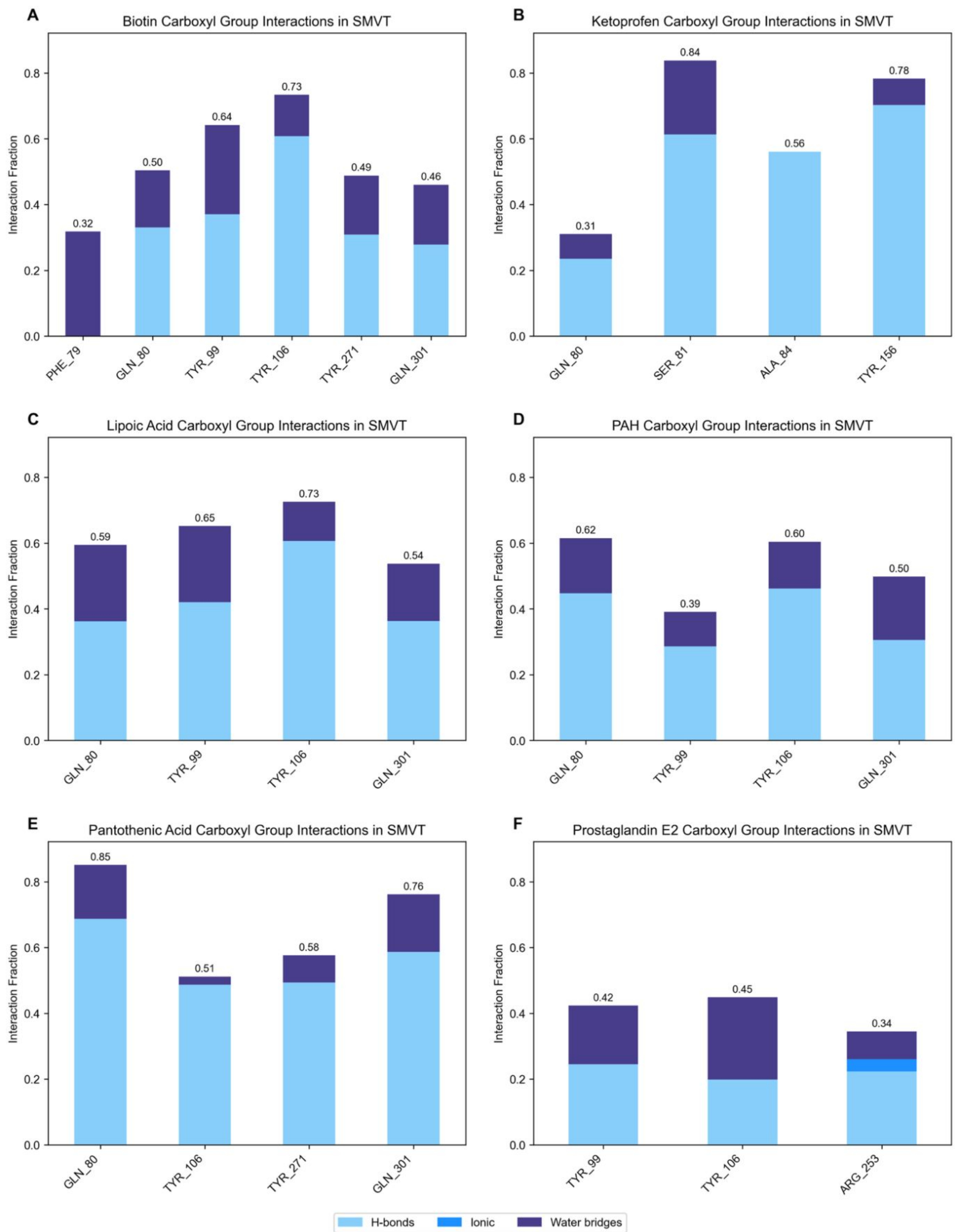

Figure S2. (A–F) Ligand carboxyl group interaction frequencies for SMVT complexes bound with selected screening compounds. Interactions shown have a frequency > 0.3, based on merged data from the five replicas where the ligand remained stable in the binding site.

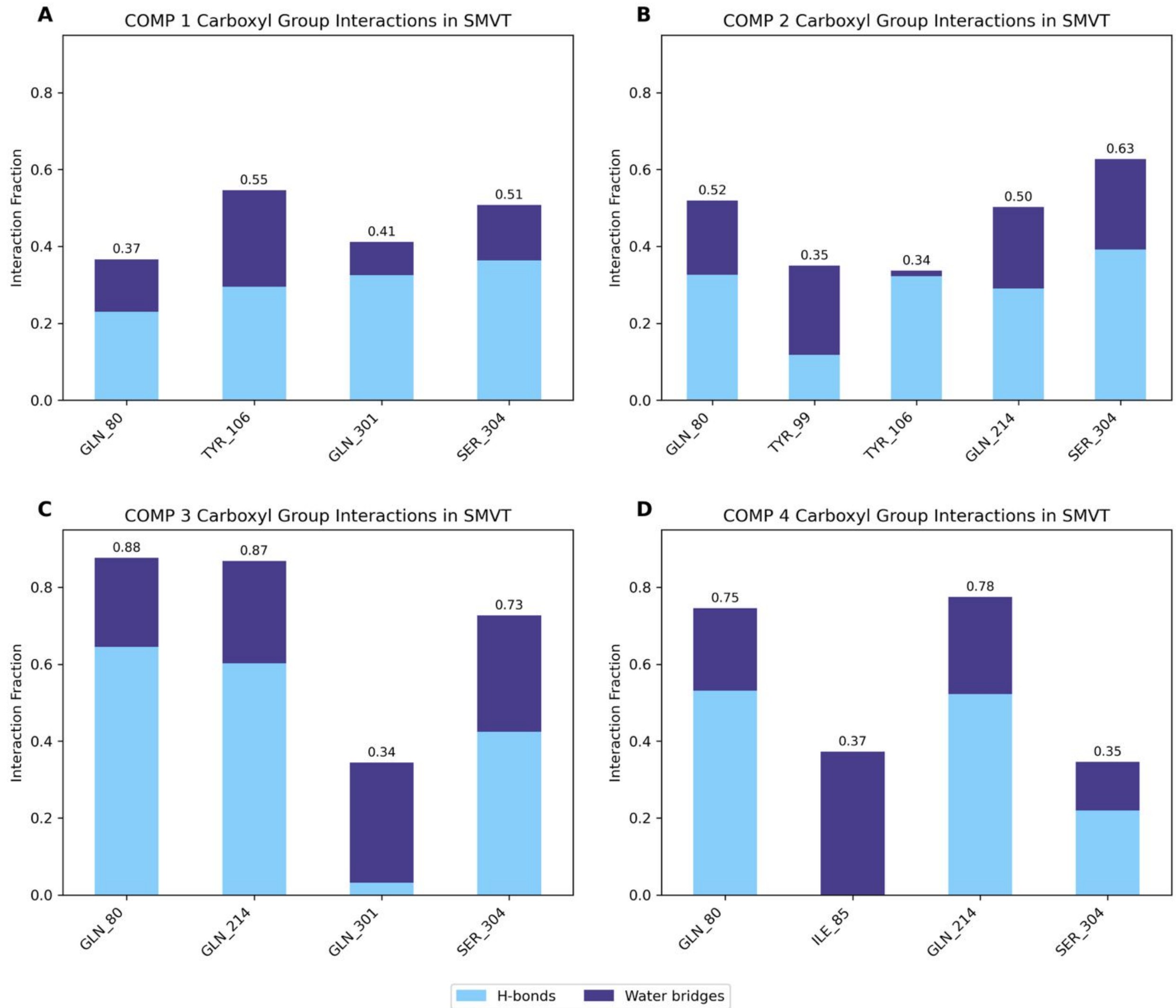

Figure S3. (A–D) Ligand carboxyl group interaction frequencies for SMVT complexes bound with COMP **1–4**. Interactions shown have a frequency > 0.3, based on merged data from the five replicas where the ligand remained stable in the binding site.

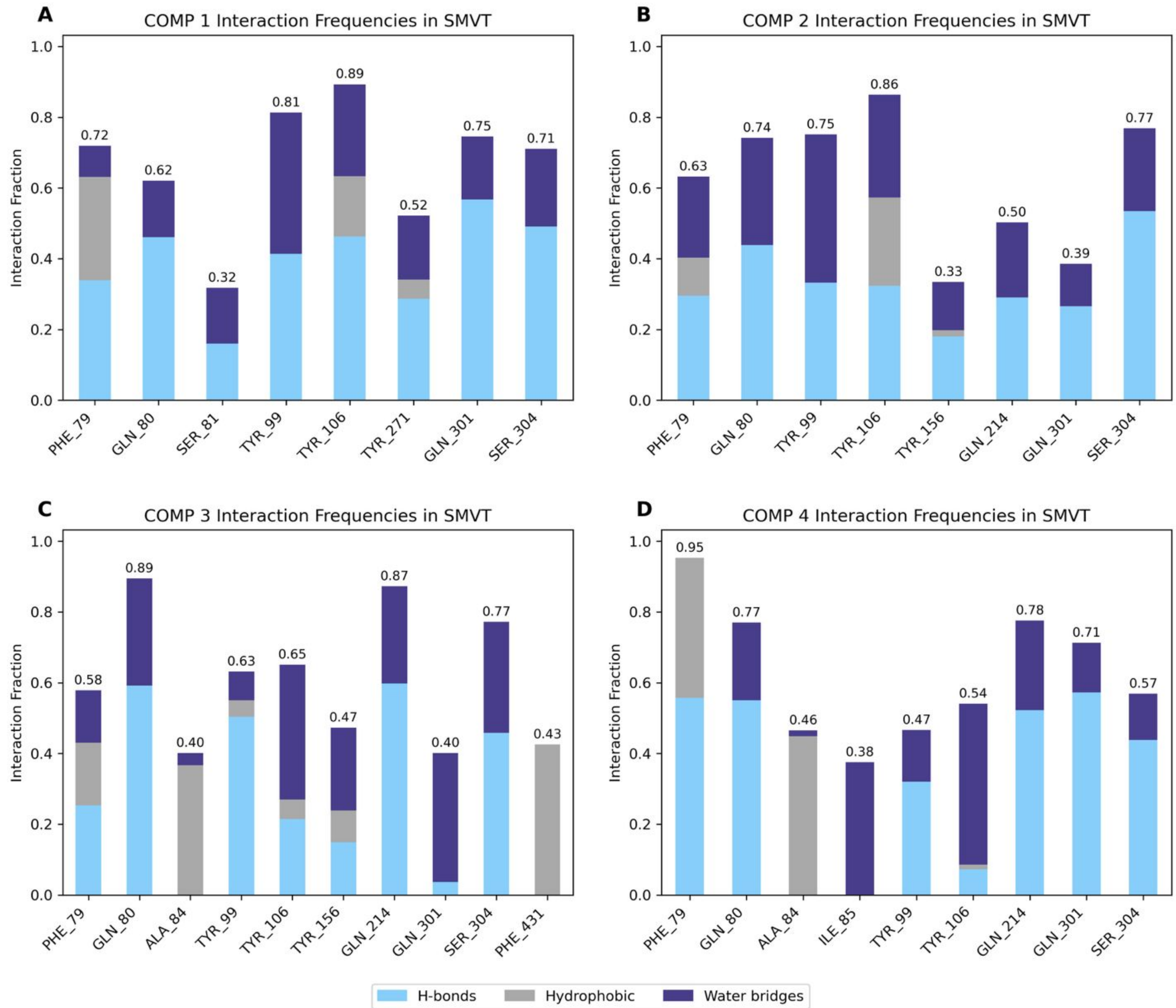

Figure S4. (A–D) Interaction frequencies for SMVT complexes bound with COMP **1–4**. Interactions shown have a frequency > 0.3, based on merged data from the five replicas where the ligand remained stable in the binding site.

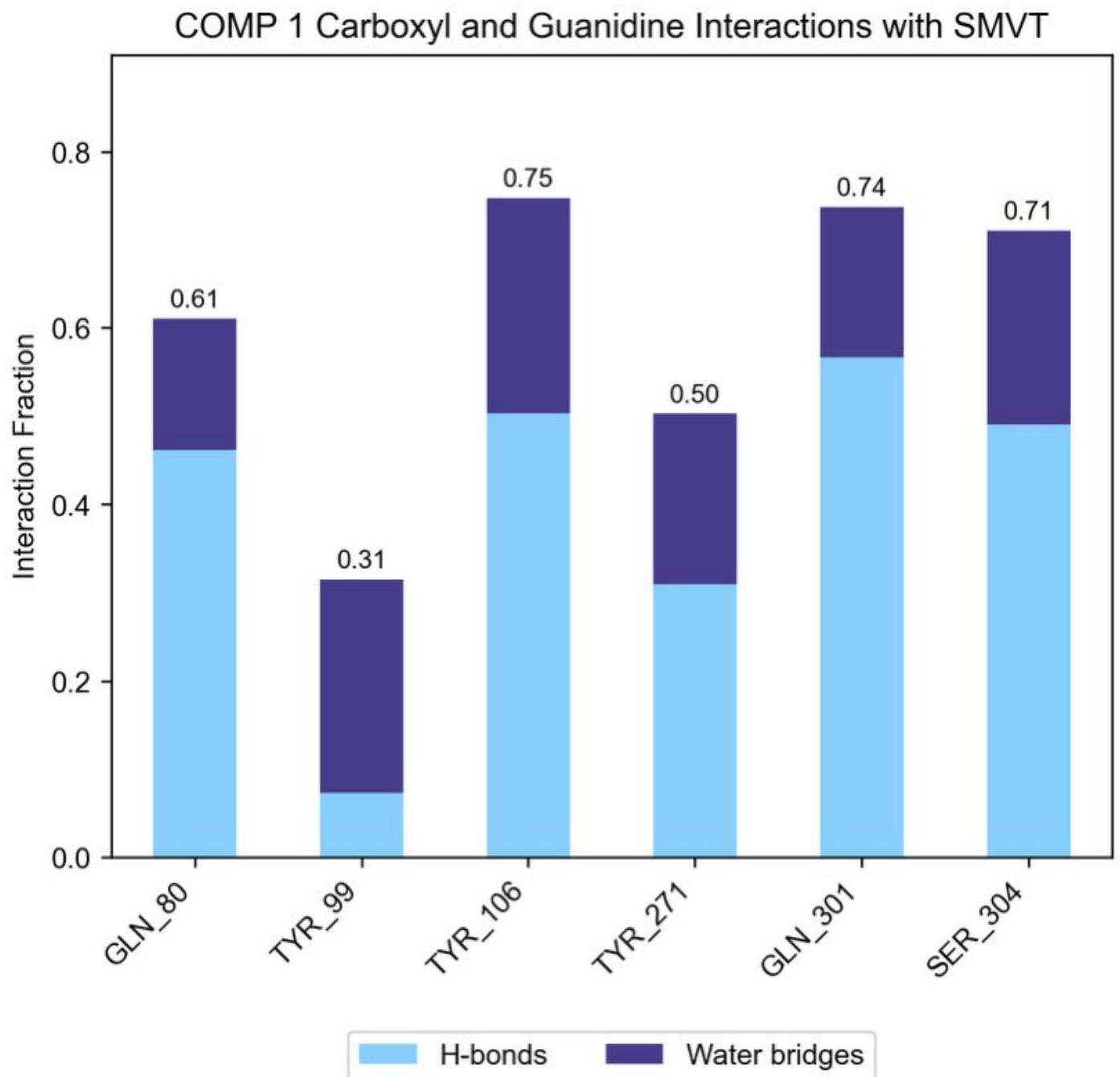

Figure S5. Combined carboxyl and guanidine group interaction frequencies for SMVT complexed with COMP **1**. Interactions shown have a frequency > 0.3, based on merged data from the five replicas where the ligand remained stable in the binding site.

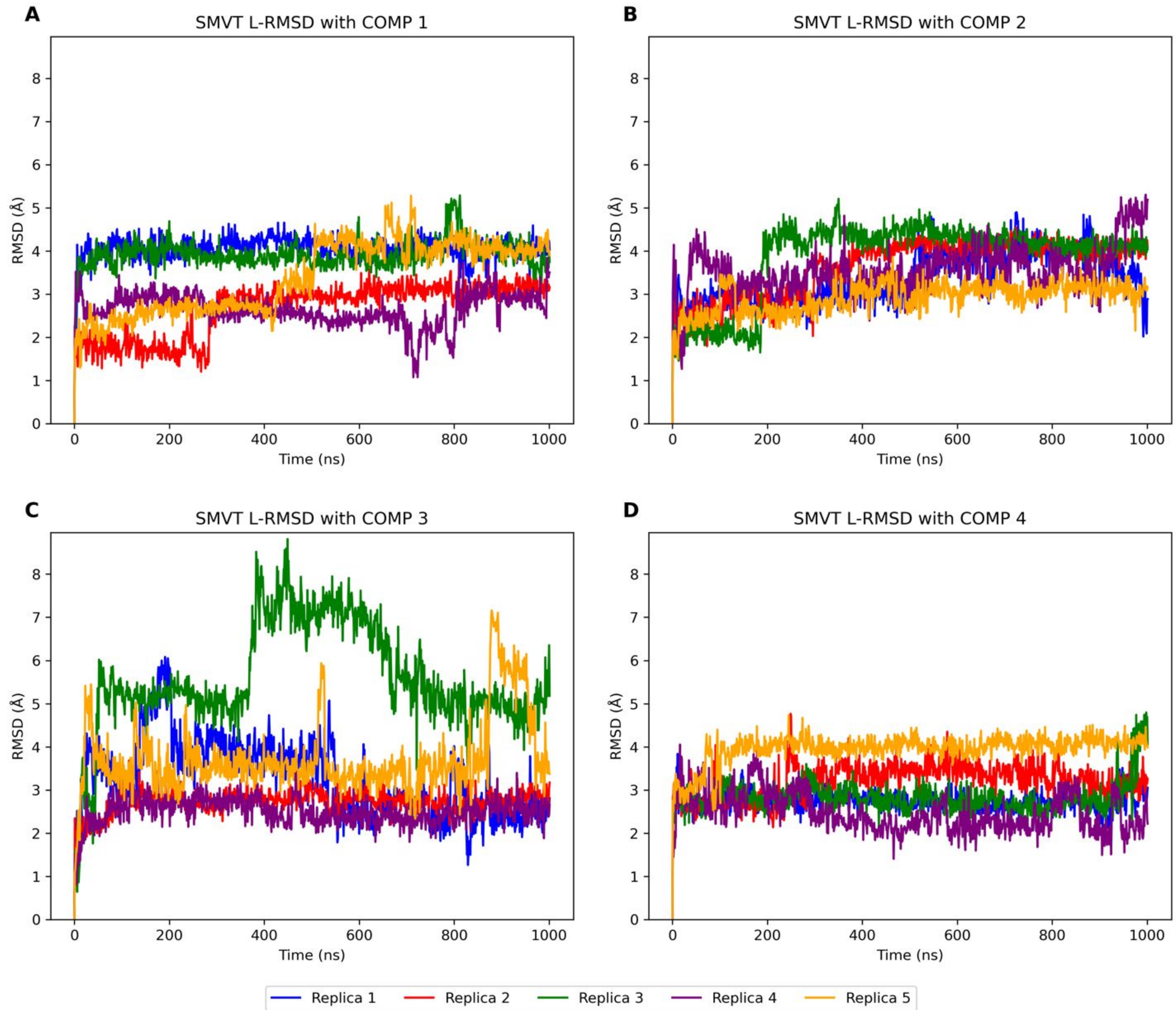

Figure S6. (A–D) The Ligand Root Mean Square Deviations (L-RMSD) for SMVT complexes bound with COMP **1–4**. L-RMSD is calculated based on the ligand heavy atoms relative to the protein-ligand complex.

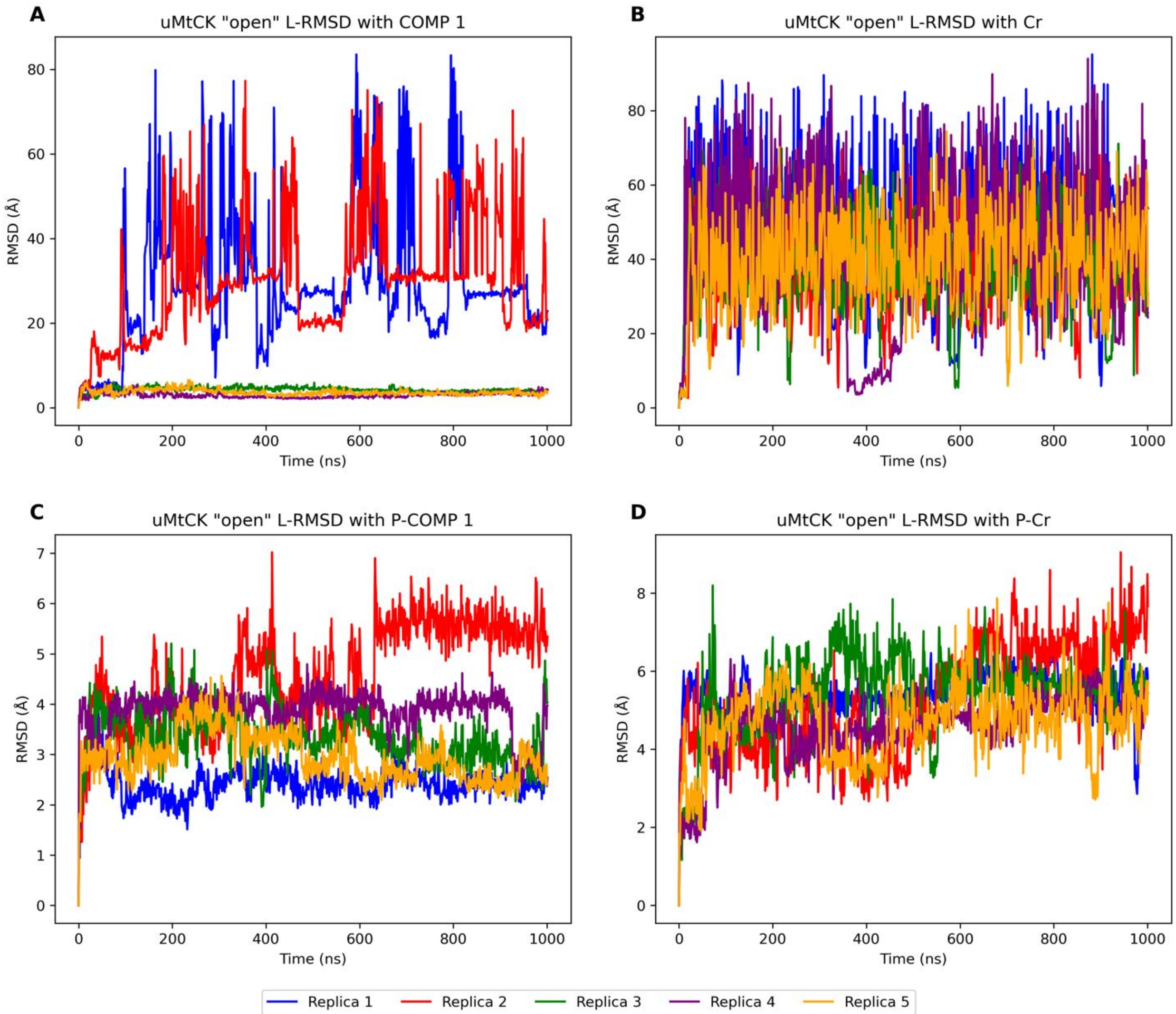

Figure S7. (A–D) The Ligand Root Mean Square Deviations (L-RMSD) for "open" uMtCK complexes bound with phosphorylated and non-phosphorylated COMP **1** and creatine. L-RMSD is calculated based on the ligand heavy atoms relative to the protein-ligand complex.

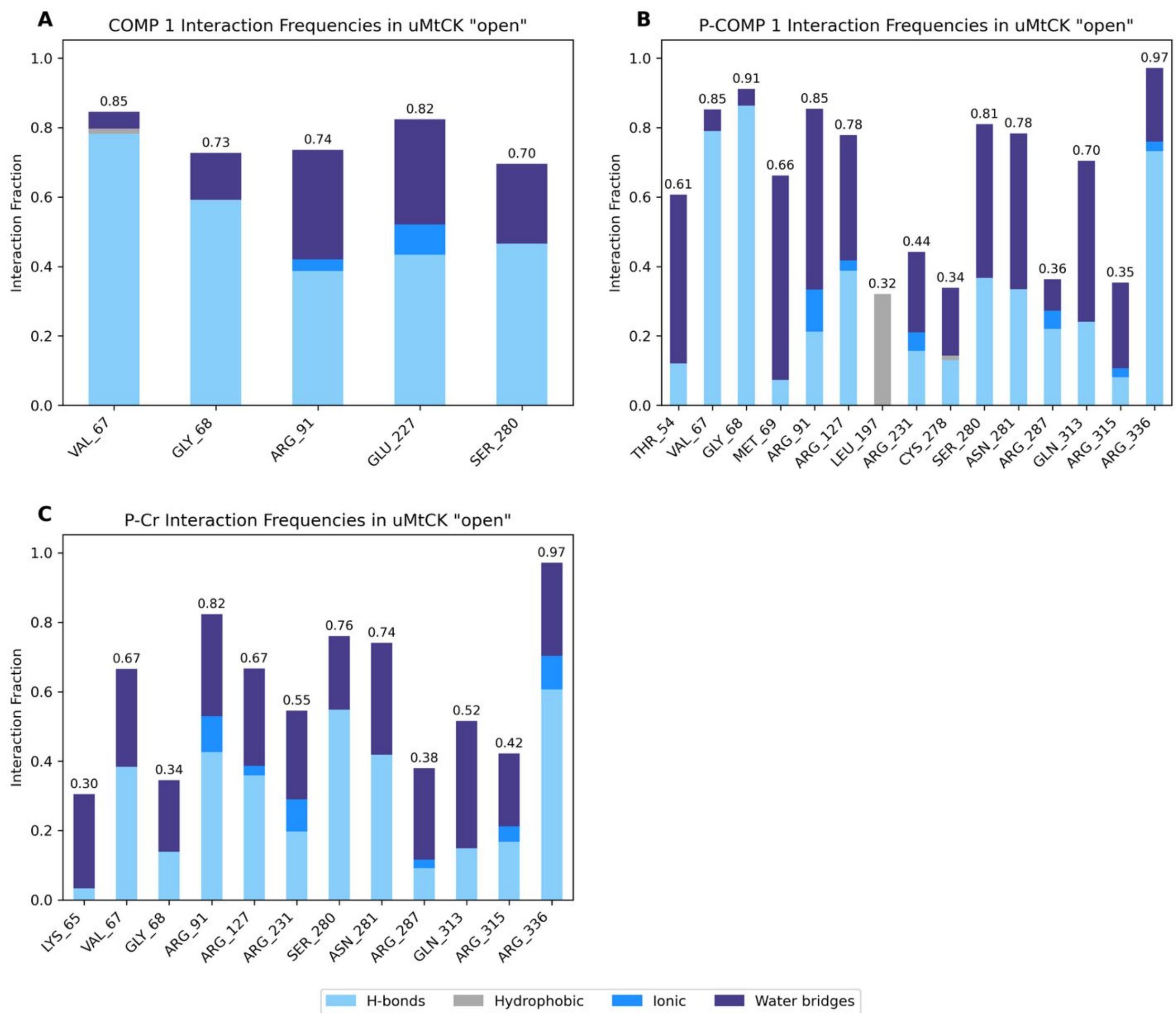

Figure S8. (A–C) Interaction frequencies for "open" uMtCK complexes bound with phosphorylated and non-phosphorylated COMP **1** and creatine. Interactions shown have a frequency > 0.3, based on merged data from the five replicas where the ligand remained stable in the binding site. Stable replicas 3-5 were used for COMP **1**.

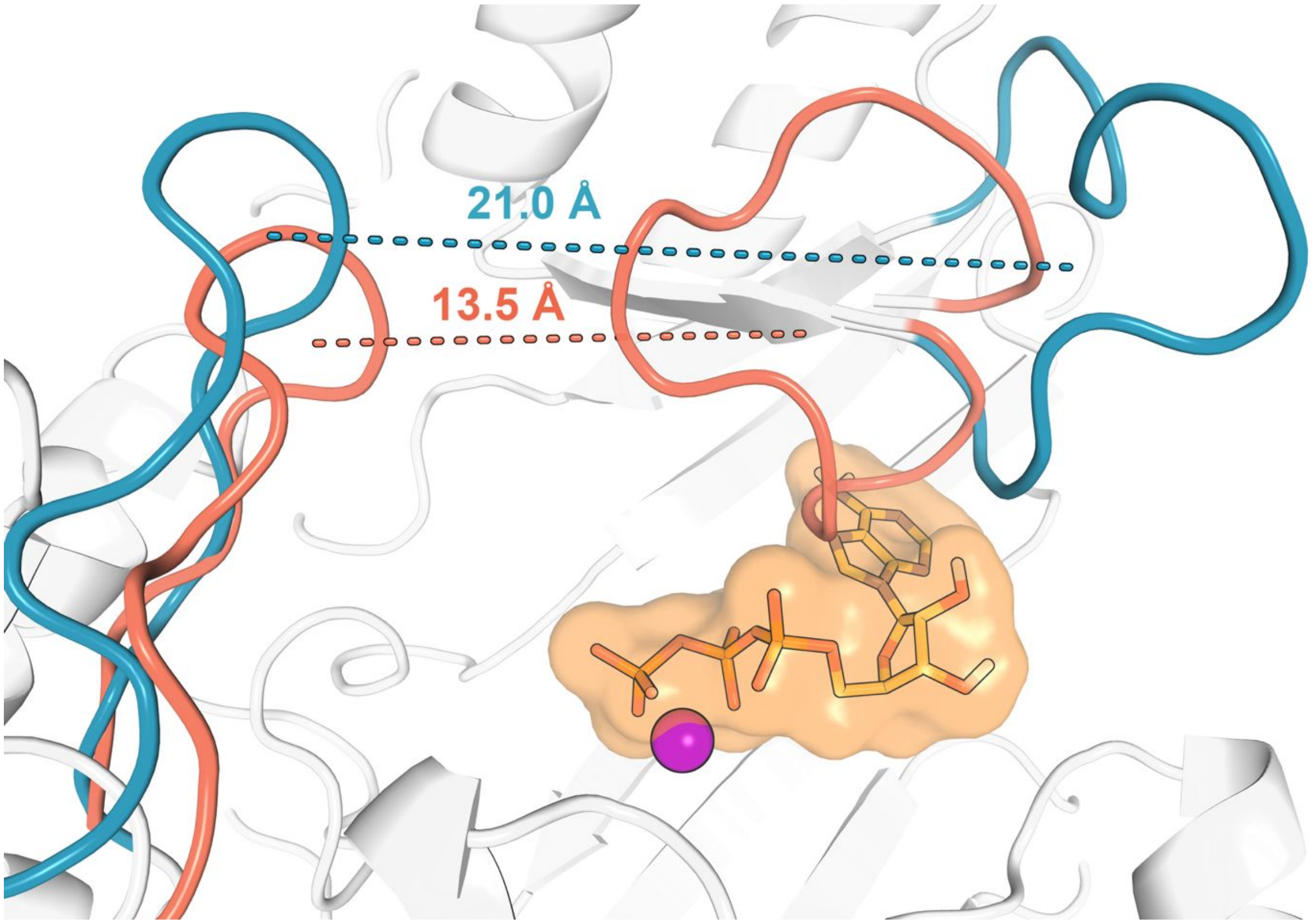

Figure S9. View of the surrounding loops near the uMtCK catalytic site, visualizing the change in loop distance during the transition from "open" (cyan) to "closed" (orange) state.

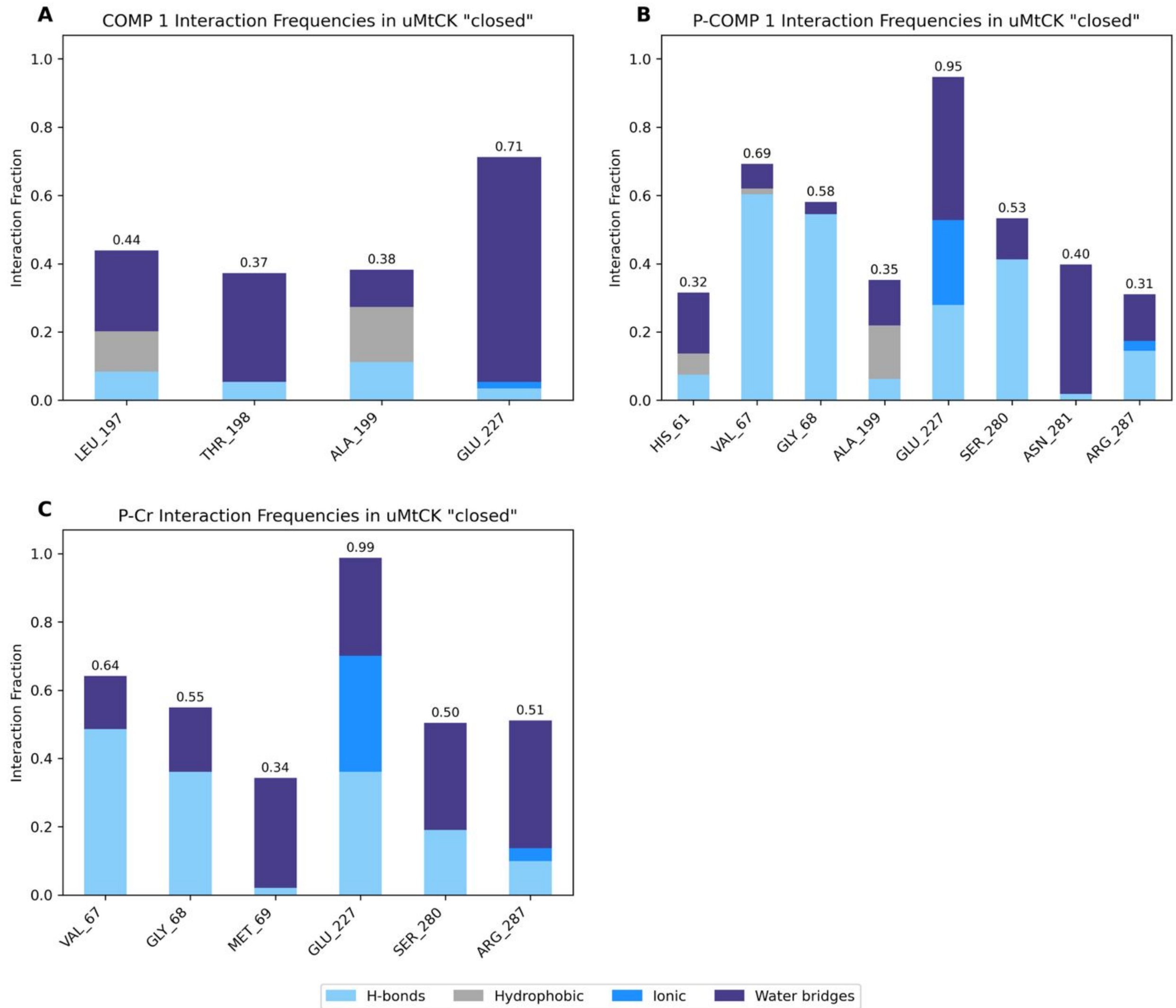

Figure S10. (A–C) Interaction frequencies for "closed" uMtCK complexes bound with phosphorylated and non-phosphorylated COMP **1** and creatine. Interactions shown have a frequency > 0.3, based on merged data from the five replicas where the ligand remained stable in the binding site.

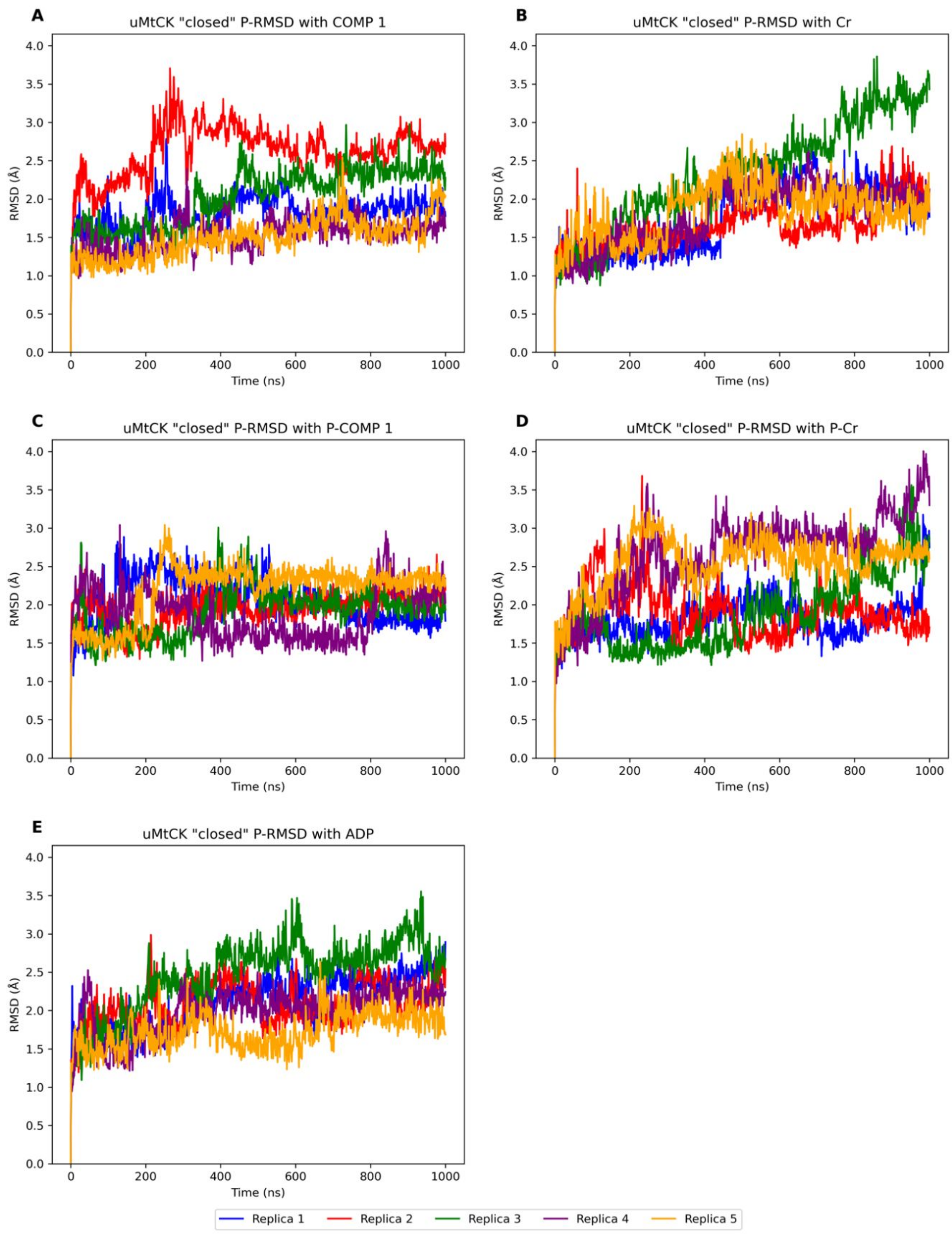

Figure S11. (A–E) The Protein Root Mean Square Deviations (P-RMSD) for "closed" uMtCK apo (ADP) and bound phosphorylated and non-phosphorylated COMP **1** and creatine complexes. P-RMSD is calculated based on the alpha carbons relative to the protein-ligand complex.

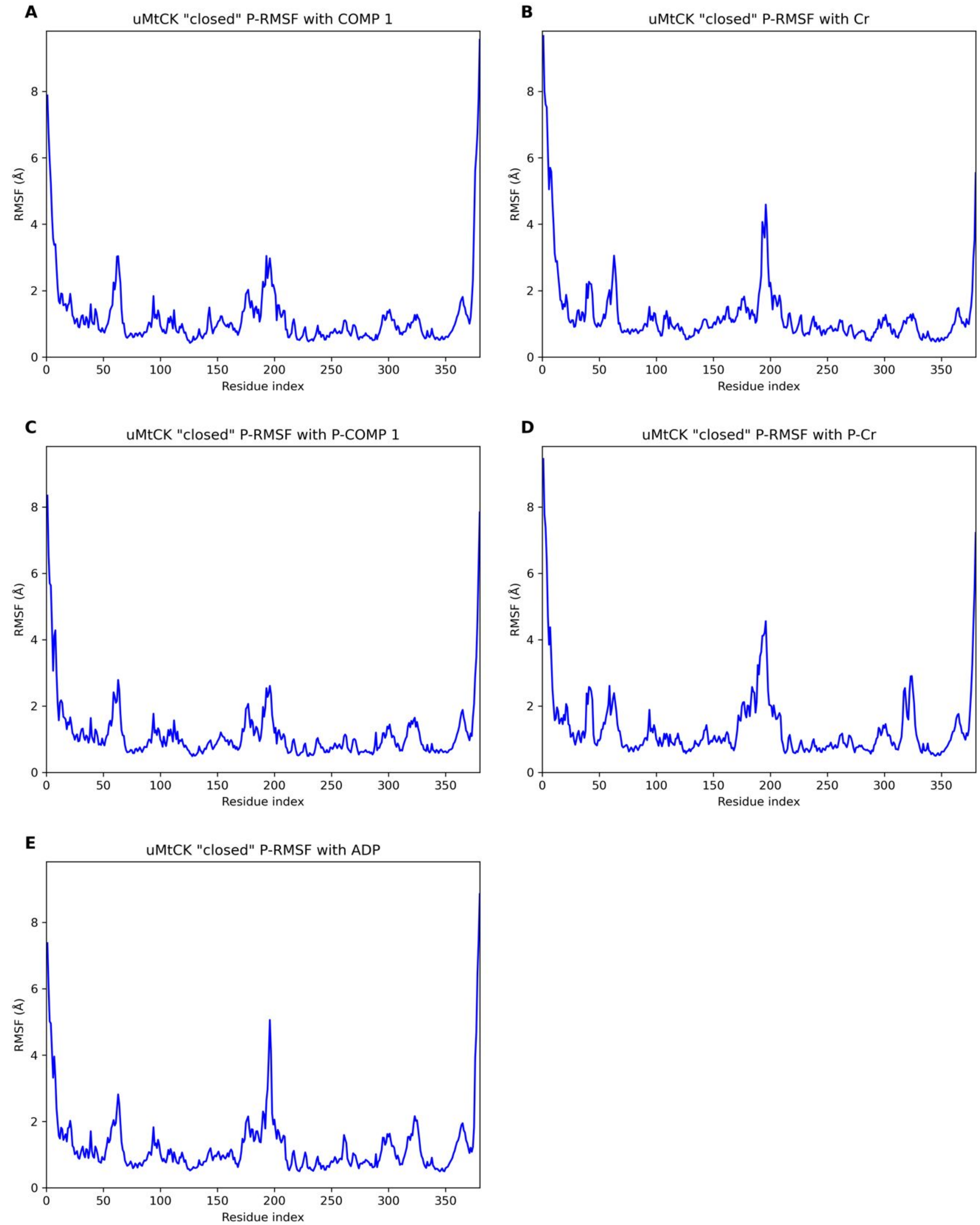

Figure S12. (A–E) The Protein Root Mean Square Fluctuations (P-RMSF) for “closed” uMtCK apo (ADP) and bound phosphorylated and non-phosphorylated COMP **1** and creatine complexes. P-RMSF represents the residue-specific average positional deviations of protein alpha carbons, with data merged from all five replicas.

Characterization of compound **1-4:**

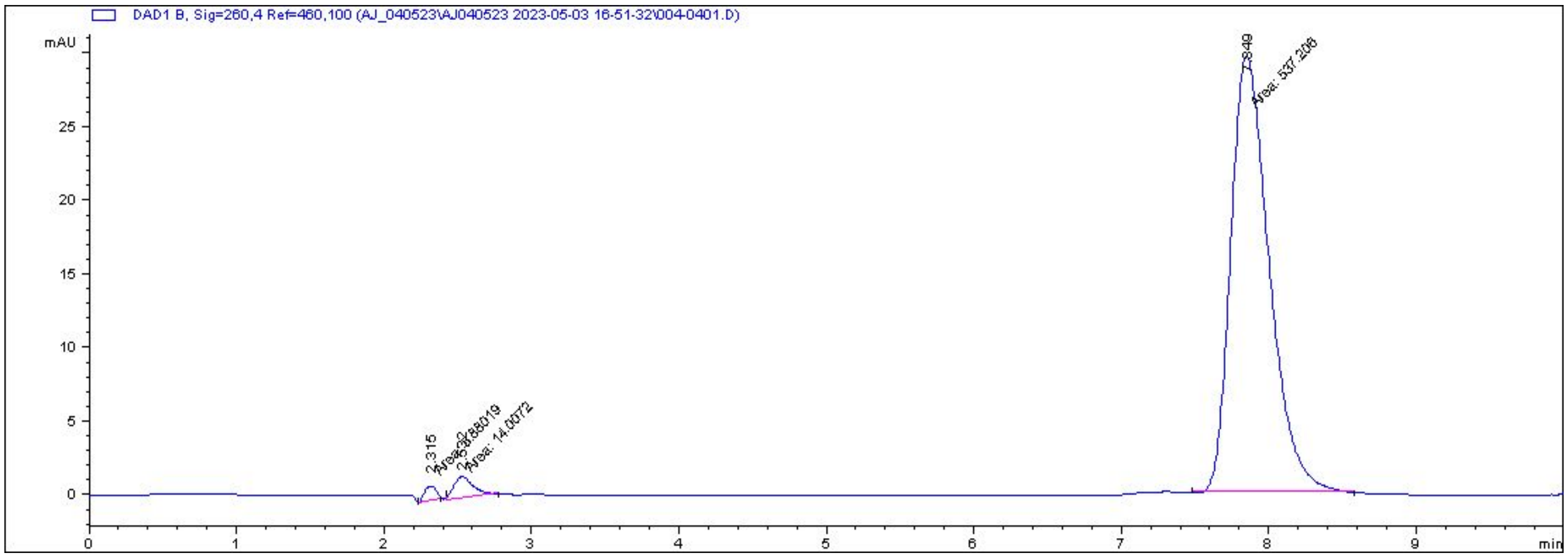

Figure S13. HPLC chromatogram of compound **1** (purity 96.43%).

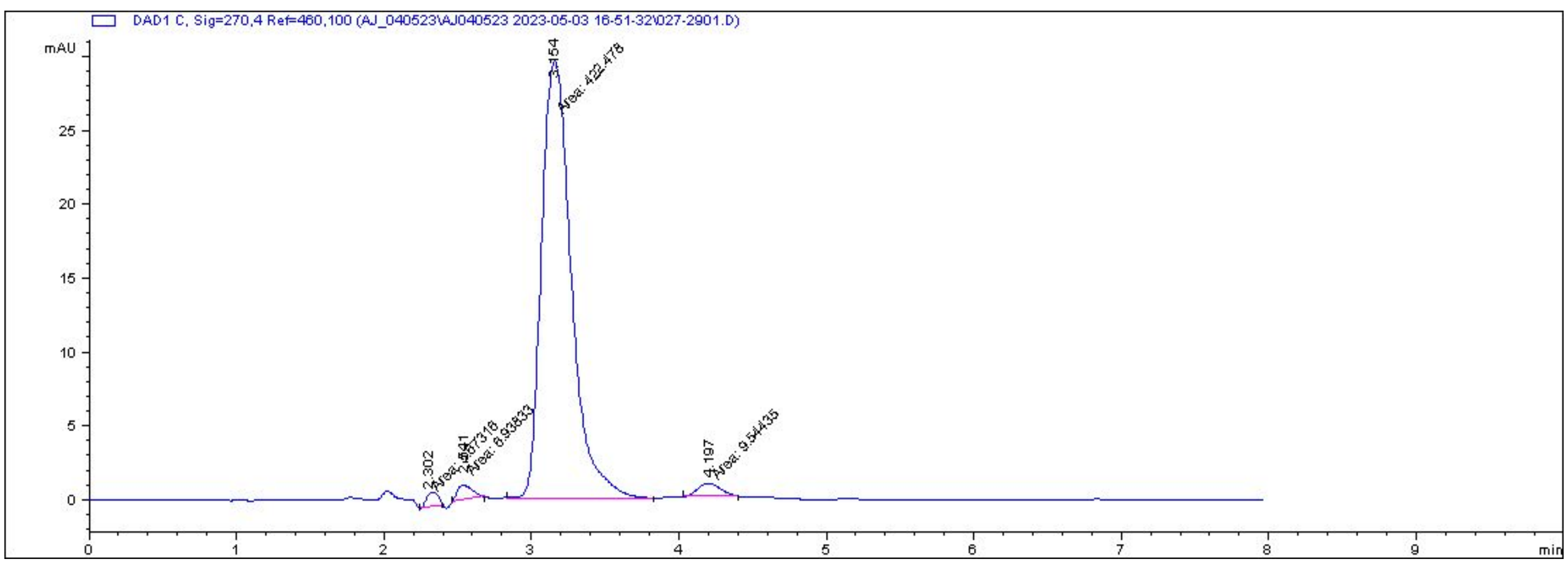

Figure S14. HPLC chromatogram of compound **2** (purity 95.02%).

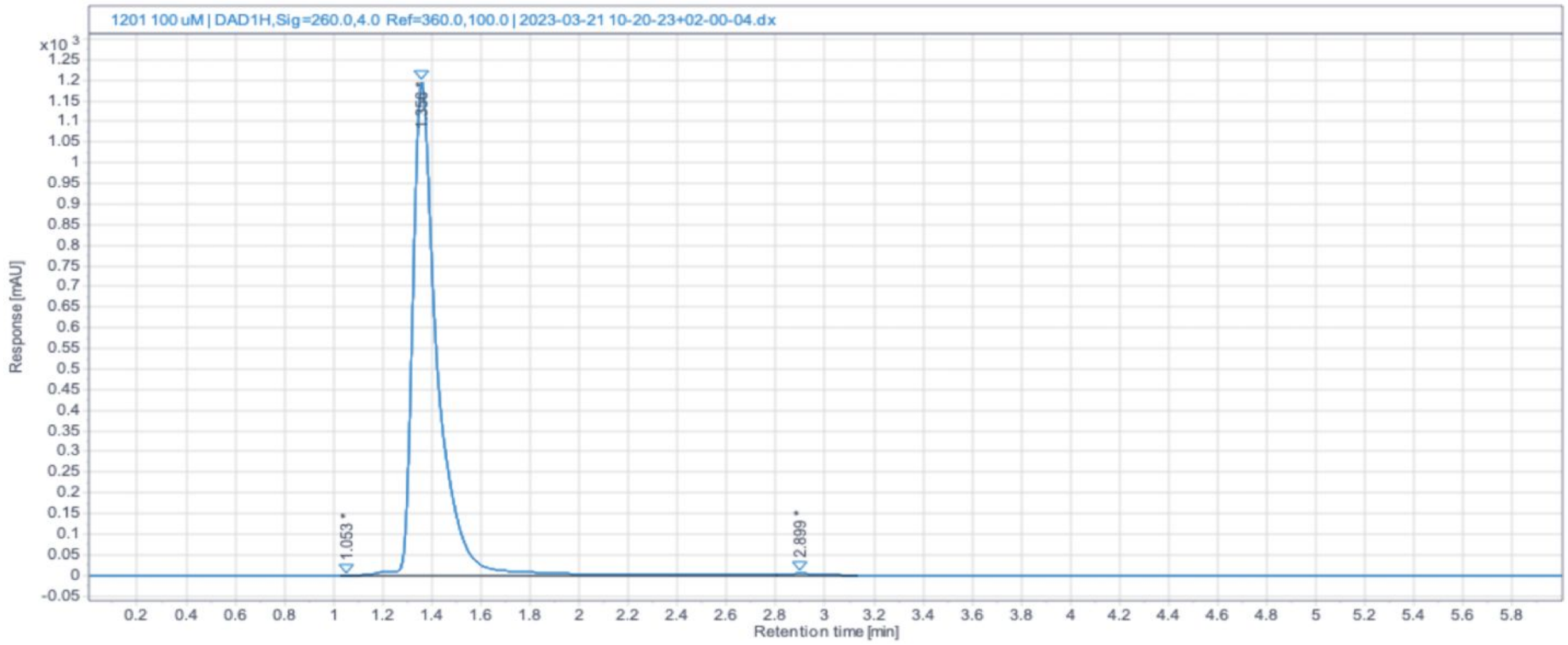

Figure S15. UHPLC chromatogram of compound **3** (purity 99.72%).

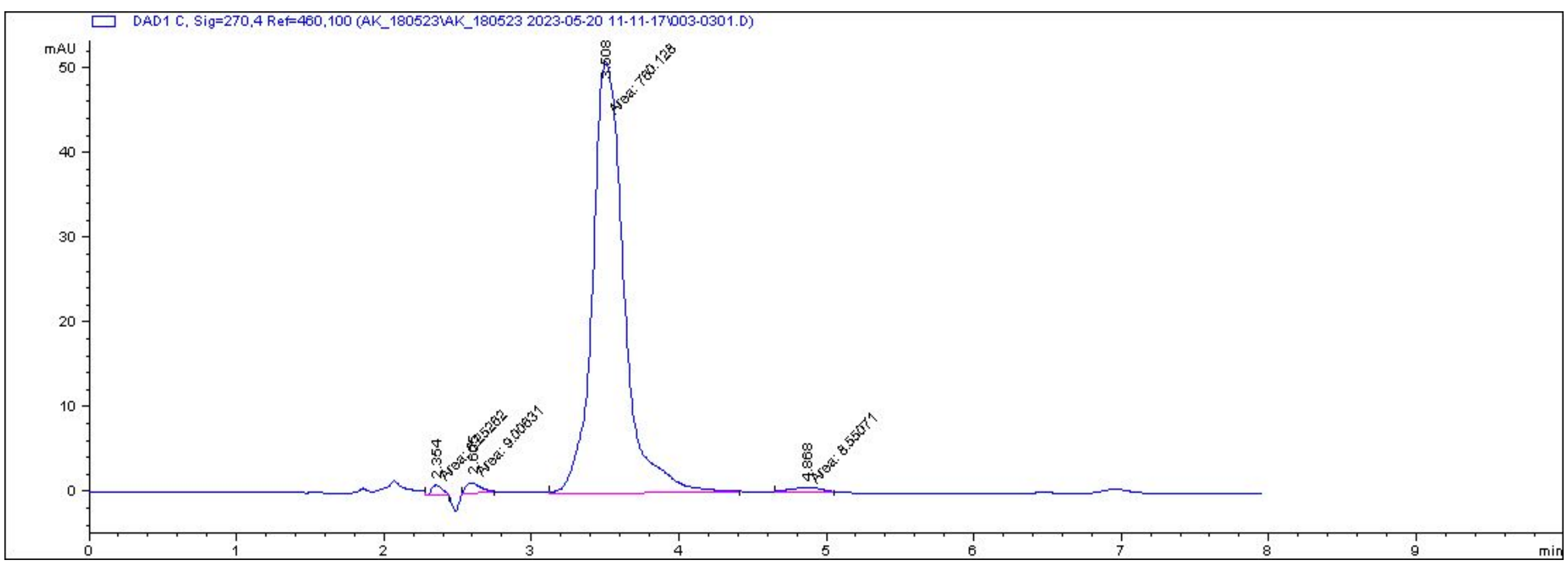

Figure S16. HPLC chromatogram of compound **4** (purity 96.96%).

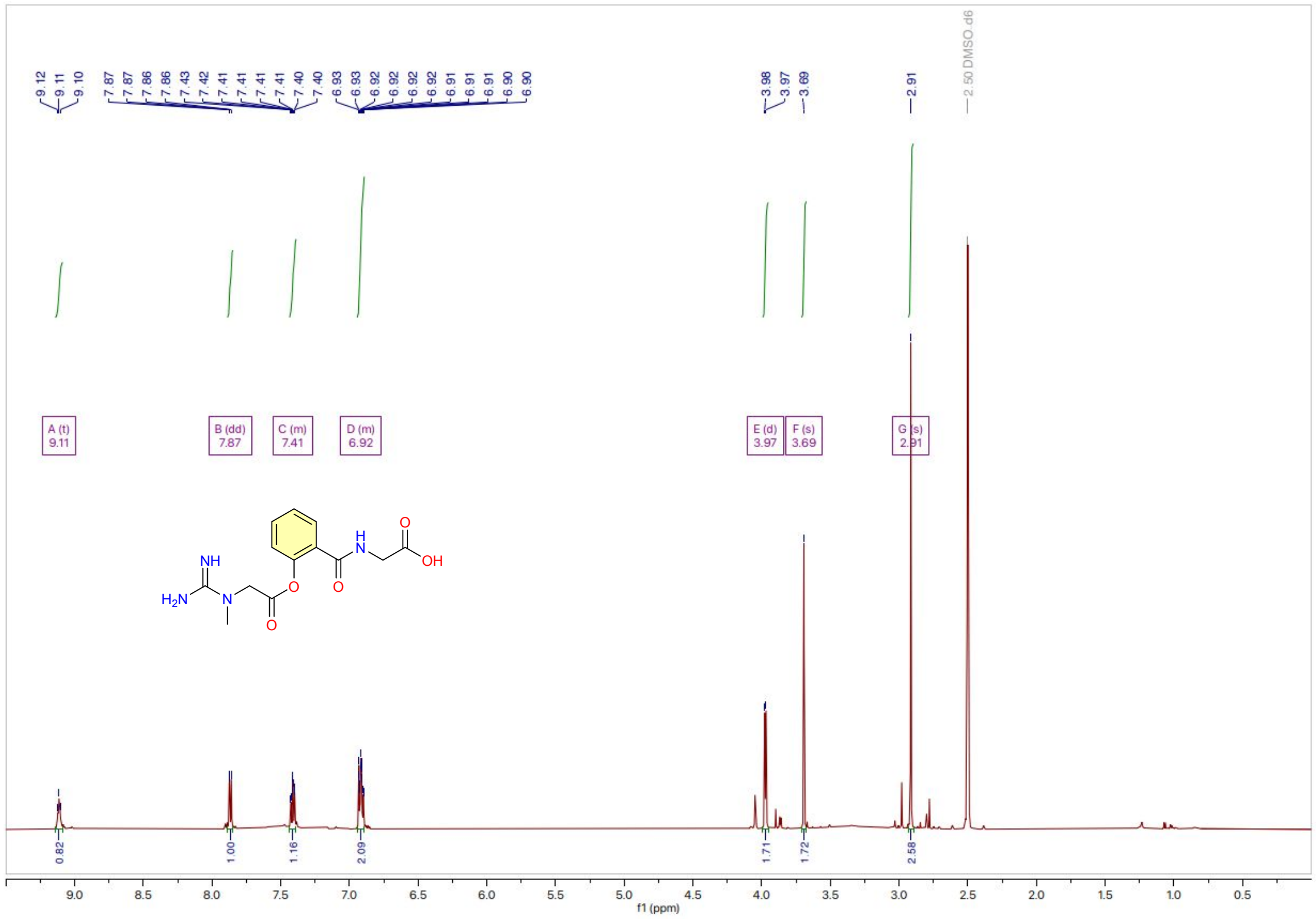

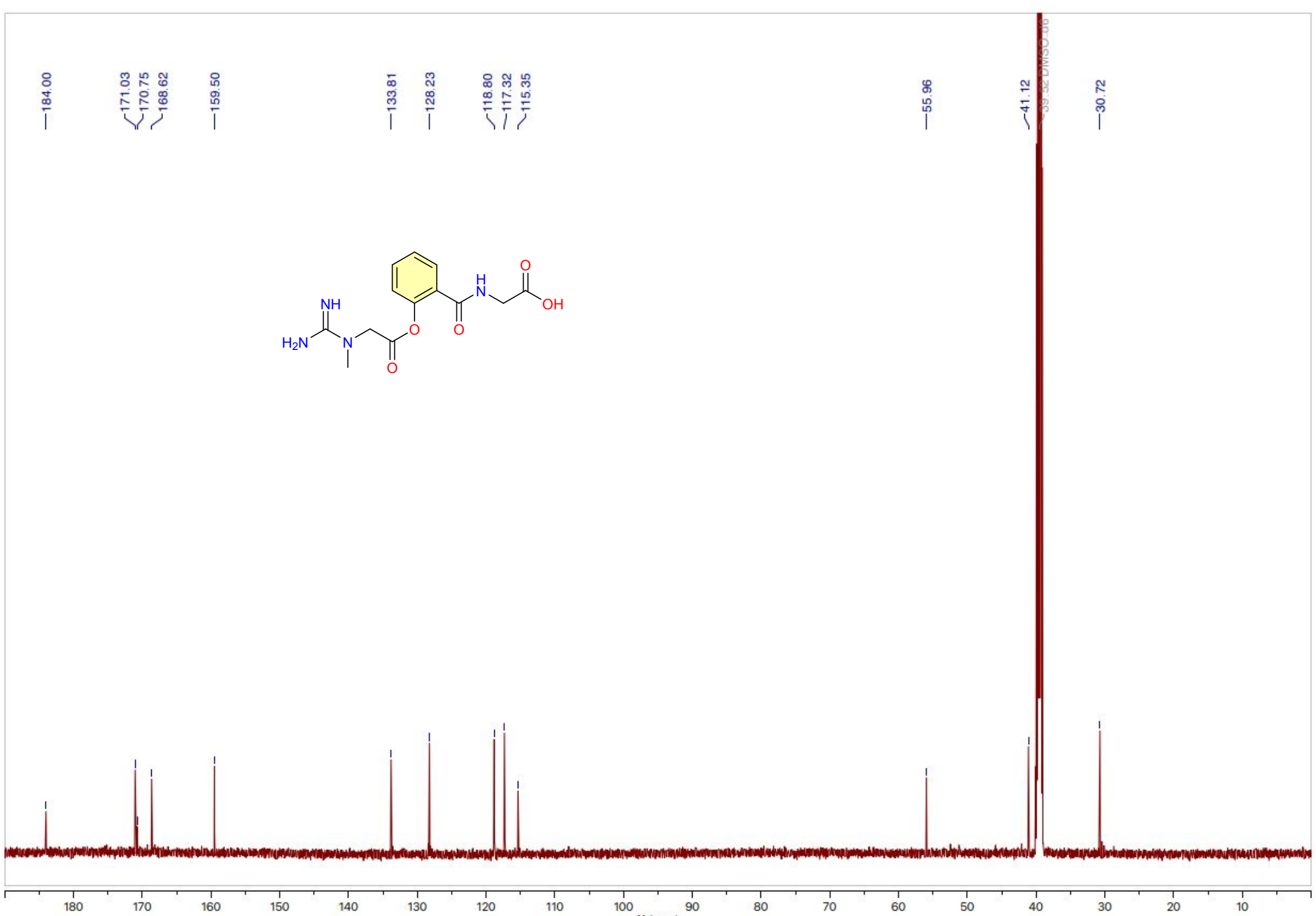

Figure S17. ^{1}H and ^{13}C NMR spectra of compound **1**.

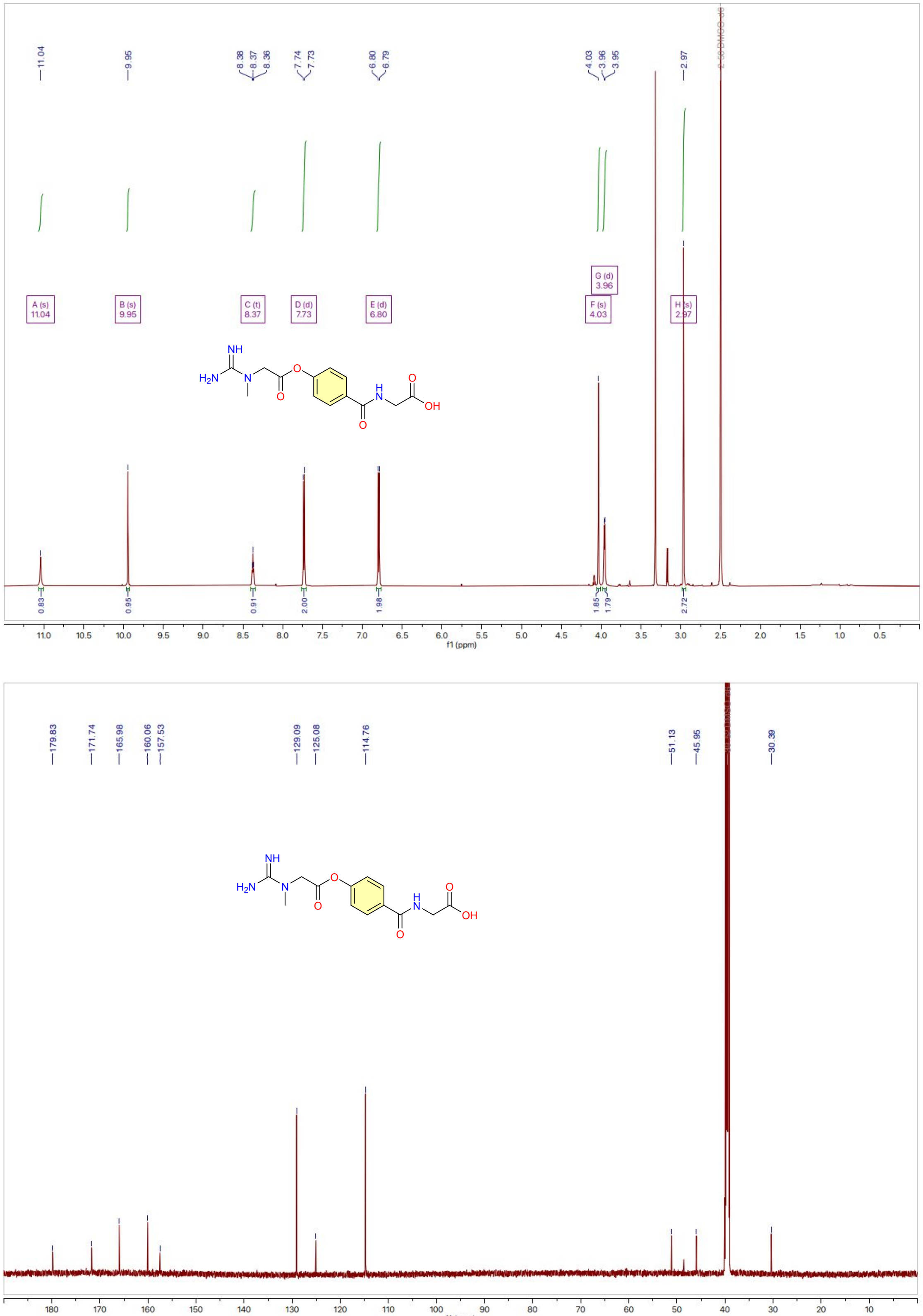

Figure S18. ^{1}H and ^{13}C NMR spectra of compound **2**.

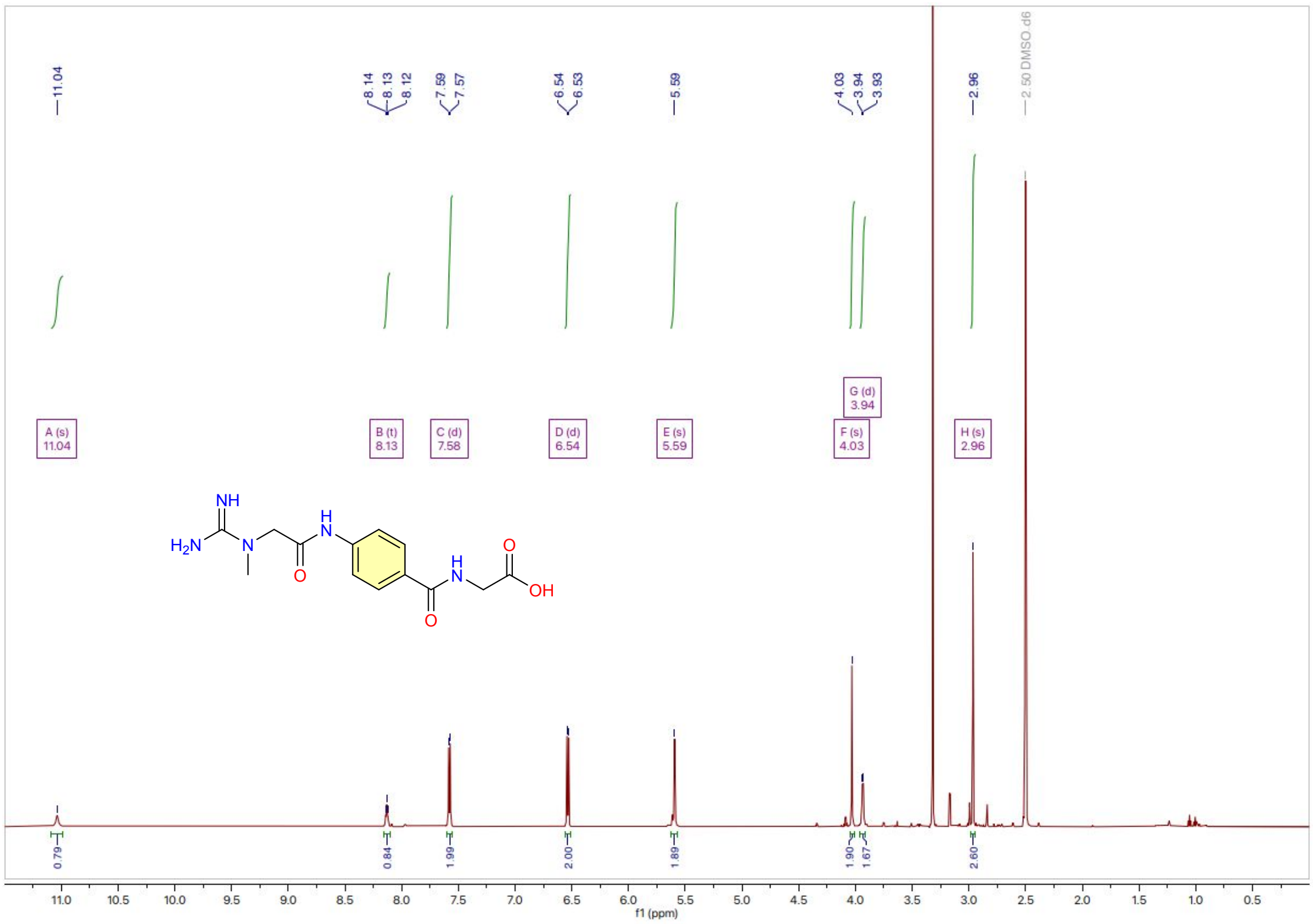

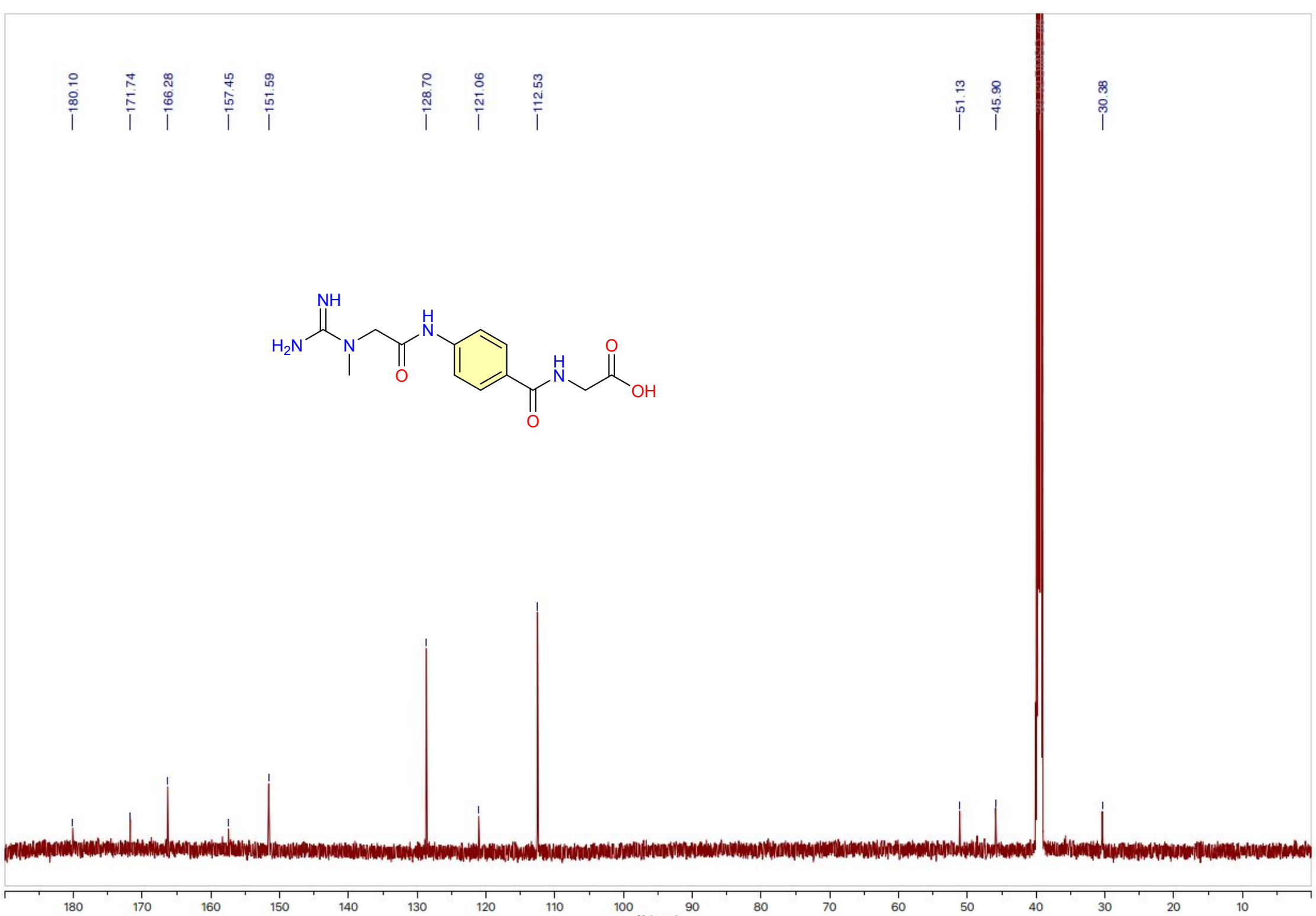

Figure S19. ^{1}H and ^{13}C NMR spectra of compound **3**.

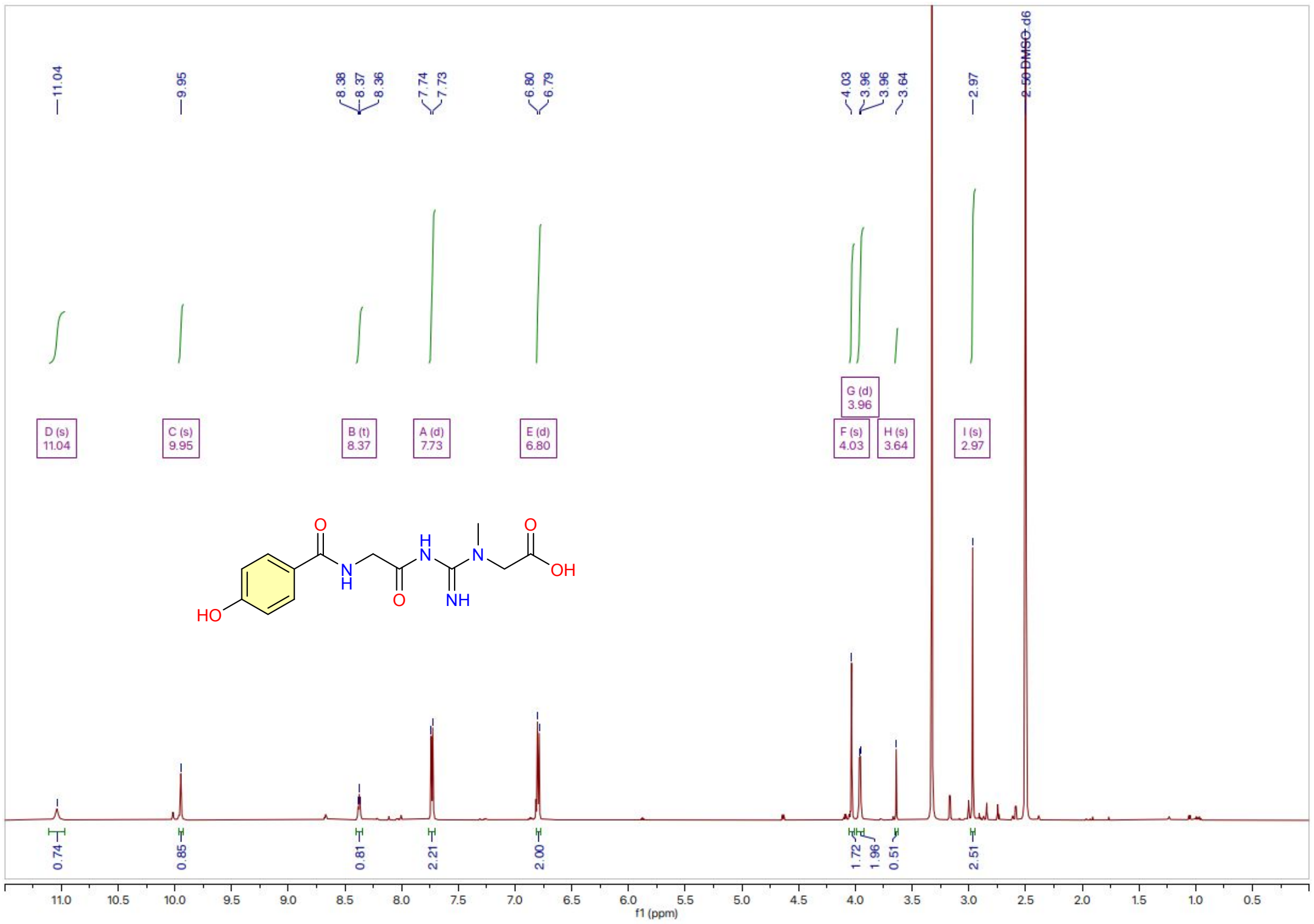

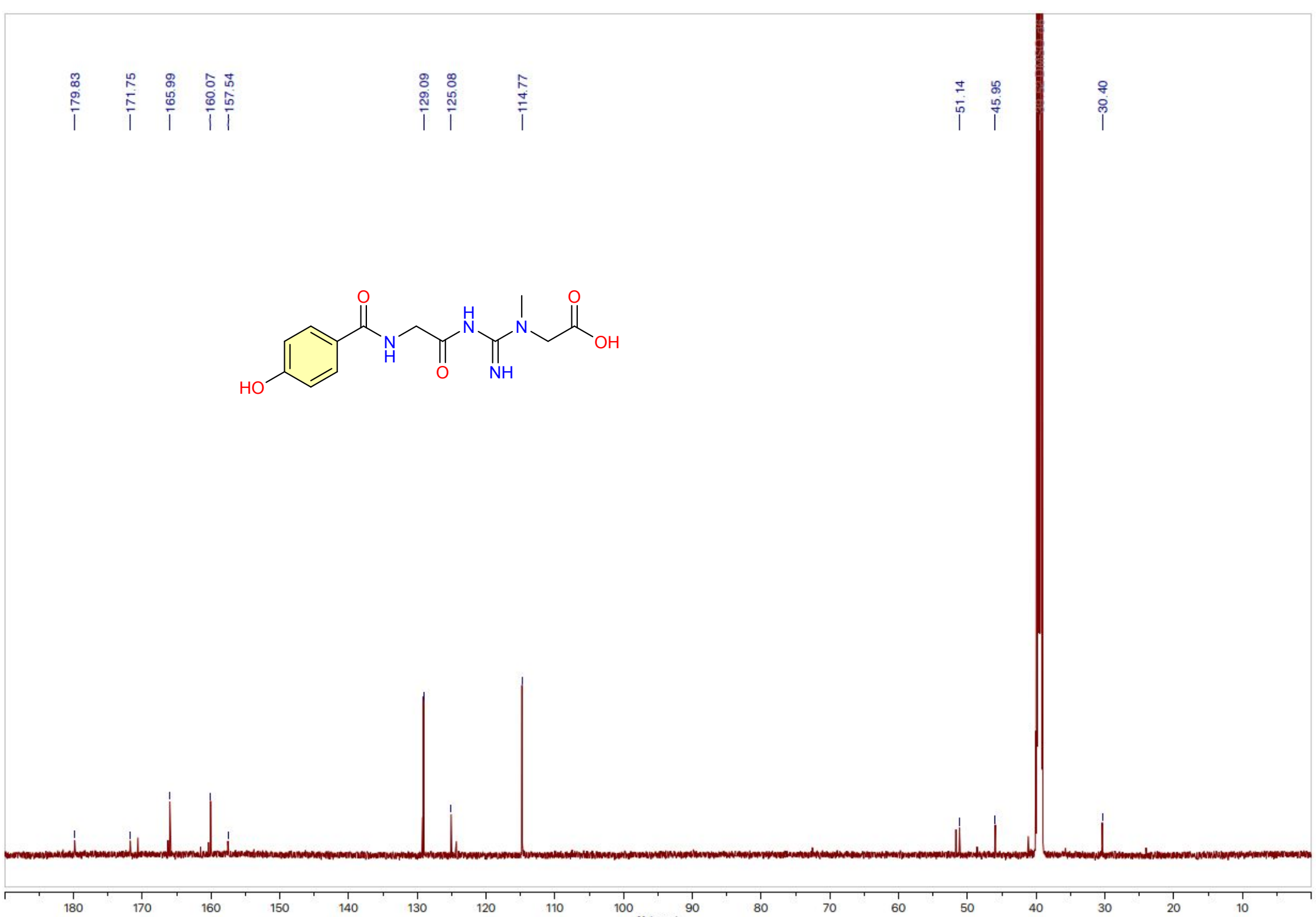

Figure S20. ^{1}H and ^{13}C NMR spectra of compound **4**.

Table S1. Transitions of the targeted LC-MS plasma membrane proteomics.

Protein	Gene	Nat/SIS	Peptide sequence	RT (min)	Precursor ion (Q1, *m/z*)	Product ions (Q3, *m/z*)
Na^+/K^+-ATPase	*Atp1a1-4*	Nat	AAVPDAVGK	10.6	414.2	586.3, 343.2, 685.4
		SIS	AAVPDA**V***GK	10.6	417.2	592.3, 346.2, 691.4
LAT1	*Slc7a5*	Nat	VQDAFAAAK	12.9	482.8	578.3, 821.4, 693.4
		SIS	VQDAFA**A***AK	12.9	486.3	825.4, 582.3, 697.4
4F2hc	*Slc3a2*	Nat	VAGSPGWVR	17.2	464.8	701.4, 614.3, 517.3
		SIS	VAGSPGWV**R***	17.2	469.8	711.4, 624.3, 527.3
SMVT	*Slc5a6*	Nat	SLNPGTIYPVLPK	35.8	699.9	599.8, 1084.6, 542.8
		SIS	SLNPGTIYPVLP**K***	35.8	703.9	603.9, 1092.7, 546.8

Nat = native peptide, SIS = stable isotope standard peptide, RT = retention time, stable labelled amino acids: **V***= Val ^{13}C(5),^{15}N(1); **A***= Ala ^{13}C(3),^{15}N(1); **R***= Arg ^{13}C(6),^{15}N(4); **K***= Lys ^{13}C(6).
